# *Mycobacterium avium subspecies paratuberculosis* (MAP) infection, and its impact on gut microbiome of individuals with multiple sclerosis

**DOI:** 10.1038/s41598-024-74975-4

**Published:** 2024-10-14

**Authors:** Hajra Ashraf, Plamena Dikarlo, Aurora Masia, Ignazio R. Zarbo, Paolo Solla, Umer Zeeshan Ijaz, Leonardo A. Sechi

**Affiliations:** 1 Department of Biomedical Sciences, University of Sassari, Sassari, Italy; 2 BIOMES NGS GmbH, Schwartzkopffstraße 1, Halle 21, 15745 Wildau, Germany; 3 Department of Medicine and Pharmacy, Neurology, University of Sassari, Sassari, Italy; 4 Water & Environment Research Group, Mazumdar-Shaw Advanced Research Centre, University of Glasgow, Glasgow, UK; 5 National University of Ireland, University Road, Galway, Ireland; 6 Department of Molecular and Clinical Cancer Medicine, University of Liverpool, Liverpool, UK; 7 Complex Structure of Microbiology and Virology, AOU Sassari, Sassari, Italy

**Keywords:** Multiple sclerosis, Gut microbiome; 16S rRNA, Mediation analysis; Neutral modelling, Microbial communities, Neurology

## Abstract

The microbial ecology of *Mycobacterium avium subspecies paratuberculosis* infections (MAP) within the context of Multiple Sclerosis (MS) is largely an unexplored topic in the literature. Thus, we have characterized the compositional and predicted functional differences of the gut microbiome between MS patients with MAP (MAP+) and without (MAP−) infection. This was done in the context of exposome differences (through self-reported filled questionnaires), principally in anthropometric and sociodemographic patterns to gain an understanding of the gut microbiome dynamics. 16S rRNA microbiome profiling of faecal samples (n = 69) was performed for four groups, which differed by disease and MAP infection: healthy cohort (HC) MAP−; HC MAP+ ; MS MAP−; and MS MAP+ . Using a dynamic strategy, with MAP infection and time of sampling as occupancy models, we have recovered the core microbiome for both HC and MS individuals. Additional application of neutral modeling suggests key genera that are under selection pressure by the hosts. These include members of the phyla *Actinobacteriota, Bacteroidota*, and *Firmicutes*. As several subjects provided multiple samples, a *Quasi Conditional Association Test* that incorporates paired-nature of samples found major differences in *Archaea*. To consolidate treatment groups, confounders, microbiome, and the disease outcome parameters, a mediation analysis is performed for MS cohort. This highlighted certain genera i.e., *Sutterella*, *Akkermansia*, *Bacteriodes*, *Gastranaerophilales*, *Alistipes*, *Balutia*, *Faecalibacterium*, *Lachnospiraceae*, *Anaerostipes*, *Ruminococcaceae*, *Eggerthellaceae* and *Clostridia-UCG-014* having mediatory effect using disease duration as an outcome and MAP infection as a treatment group. Our analyses indicate that the gut microbiome may be an important target for dietary and lifestyle intervention in MS patients with and without MAP infection.

## Introduction

Multiple sclerosis (MS) is a globally prevalent neurological autoimmune disease that affected around 2.8 million people worldwide in 2020, with a higher incidence in women^1^, although recently, this figure is reported as 1.8 million^2^. Despite extensive research, the exact etiology of MS remains partly unknown, with evidence pointing to a complex interplay of genetic, lifestyle and environmental factors^3^.

Beyond this, the emerging field of the gut microbiome has shown that the human gut harbours a diverse community of bacteria, archaea, eukaryotes, viruses and other microorganisms whose composition and functionality vary in both health and disease^4,5^. The factors that influence the gut microbiome are multifactorial and both intrinsic (e.g. age, gender, genetics, disease) and extrinsic (diet, location, environmental conditions, physical activity, medications, etc.). This and the growing evidence of the gut-brain connection, often referred to as the gut-brain axis, has increased interest in exploring the dynamics of gut microbiome composition and function in MS patients as a potential target for MS disease treatment^6,7^. Several studies have already demonstrated the important role of the gut microbiome in the pathogenesis of MS^3,8,9^.

On the other hand, several studies have linked Mycobacterium avium subspecies paratuberculosis (MAP) infections with MS^4,5^. MAP is a versatile intracellular parasite that colonises intraepithelial macrophages in the mucosa-associated lymphoid tissue (MALT) of the small intestine. It can induce chronic granulomatous gastroenteritis, known as John’s disease or paratuberculosis, in animals, especially ruminants^6^. Infected animals can transmit the pathogen to humans in various ways, e.g. through the excretion of the bacterium in faeces, meat or milk and the subsequent consumption of contaminated animal products by humans. While the pathogen can persist in animals for years without causing disease^7,8^, various molecular and serological tests have reported the presence of MAP in the blood of individuals with multifactorial diseases, including type 1 diabetes (T1D)^9,10^, Crohn’s disease (CD)^11^, multiple sclerosis (MS)^4,5^ and Parkinson’s disease (PD)^12^. The pathogen was also isolated from intestinal mucosa biopsies of patients with Crohn’s disease and irritable bowel syndrome^13^. Molecular mimicry is known to be one of the potential mechanisms by which MAP triggers autoimmune diseases due to the structural similarity of MAP antigens to self-antigens^14^. Particularly studies in the Sardinian population, a region with a high prevalence of MS in the local population^15^, have associated MAP infections to increased susceptibility to MS, emphasising their possible role in the disease etiology^14,16,17^.

To our knowledge, no study has yet established a link between MAP infection and the gut microbiome in the context of MS. Since the pathogen resides in the gut, the way it interacts with the gut microflora is of great interest. To address this scientific question, we conducted an observational cross-sectional study in which the taxonomic composition and predicted function of the gut microbiome of MAP-positive and MAP-negative MS patients was analysed and compared with healthy controls to understand the dynamics of the gut microbiome in MS patients with and without MAP infection and to identify targets for the gut microbiome that can be treated through lifestyle and diet.

## Materials & methods

### Ethical approval and consent

This study was approved by the Ethics Review Board at University of Sassari at Azienda Sanitaria Locale (ASL) 1 (Prot.^llo^ N°2150/CE, 17/02/15). All participants provided written informed consent to participate in the study and a self-reported detailed questionnaire recording participant’s medical history, dietary habits, sleeping pattern and routine lifestyle. All methods were performed in accordance with the relevant guidelines and regulations.

### Study participant identification and recruitment

The study was conducted from early 2021 to mid-2022 in Sassari, Italy. It comprised three parallel groups for comparison: MS patients who tested positive for MAP, MS patients who tested negative for MAP and a control group consisting of healthy individuals. Two stool samples T1 and T2 were provided by each individual, typically a month apart. A total of 97 individuals were screened for participation in this study at the Multiple Sclerosis Center of the University of Cagliari, Italy. All participants underwent screening for MAP infection. This screening process involves the presence of antibodies against a MAP antigen, which was detected using the Enzyme-Linked Immunosorbent Assay (ELISA). This method has already been discussed elsewhere^10,18^.

When selecting the participants, particular effort was made to ensure no statistically significant differences between the cohorts in terms of gender, age, ancestry, BMI, type of diet (omnivore, pescetarian, vegetarian, vegan) and environmental conditions. The healthy control group came from the same geographical region as the MS patients, and followed similar criteria as above, except age. Additionally, it was ensured there were no active infectious diseases (with the exception of MAP for the MAP positive MS cohort).

Of the 97 stool samples collected from the participants, only 74 had sufficient DNA yield to carry out microbiome analyses resulting in samples originating from 51 unique participants, with further 5 dropping due to low read numbers (< 5000 reads), thus totaling 69 samples (originating from 49 unique participants) that made it to the final analyses with their details provided in the Supplementary Tables S1–S13. Note that the participant-wise statistics are calculated for 49 unique participants.

### Samples processing

The stool samples of the participants were collected using the INTEST.pro kit from BIOMES NGS GmbH, Wildau, Germany. The kit contains a sterile swab, a tube with a DNA preservation solution for the stool sample and an envelope for return transport. Participants were instructed to take a pinhead-sized stool sample from the used toilet paper with the sterile swab and then dip it into the preservative liquid in the tube. After sampling, the samples were sent to the laboratory of BIOMES NGS GmbH, Wildau (Germany). The samples were stored at room temperature between sampling and analysis. Prior to laboratory analysis, each sample was registered and activated on the company’s online portal.

After collection and activation, the faecal samples were sent to the BIOMES NGS GmbH laboratory (all between autumn 2022 and spring 2023). At the laboratory, the samples were first lysed to break up all the cells. The microbial genomic DNA from faecal material was extracted using the bead beating technique. As the most promising primer pairs for bacterial and archaeal primers ^19^, the V3-V4 region of the 16S rRNA gene was amplified and sequencing was performed on the Illumina MiSeq platform using a 2 × 300 bp paired-end protocol according to the manufacturer’s instructions (Illumina, San Diego, CA, USA).

### Bioinformatics and statistics

We have obtained 4,244,282 paired-end reads from 74 samples. On these, we have recovered representative Operational Taxonomic Units (OTUs) at 99% similarity using the same approach as used previously^20^ with the modifications: (a) we have used the recent SILVA SSU Ref NR database release v.138^21^; and (b) we generated the rooted phylogenetic tree within the QIIME2 framework^22^. Furthermore, we used PICRUSt2^23^ within the QIIME environment to recover KEGG enzymes (10,543 enzymes for 74 samples) and MetaCyc pathway (487 enzymes for 74 samples) predictions for all the samples. For this purpose, we have used the parameters –p-hsp-method pic –p-max-nsti 2 in qiime picrust2 full-pipeline [https://github.com/picrust/picrust2/wiki/q2-picrust2-Tutorial/]. QIIME2 was also used to generate a final BIOM file that combined abundance information with the new taxonomy with a final n = 74 × P = 17,164 OTUs abundance table, and which along with the newly phylogenetic tree, and the meta data was used for the downstream statistical analysis, with details given in the supplementary material. Note that for the downstream statistical analyses, the viable samples were reduced from n = 74 to n = 69 samples after removing samples where read counts per sample < 5000 reads.

## Results

### Diversity measures

**Fig. 1:**
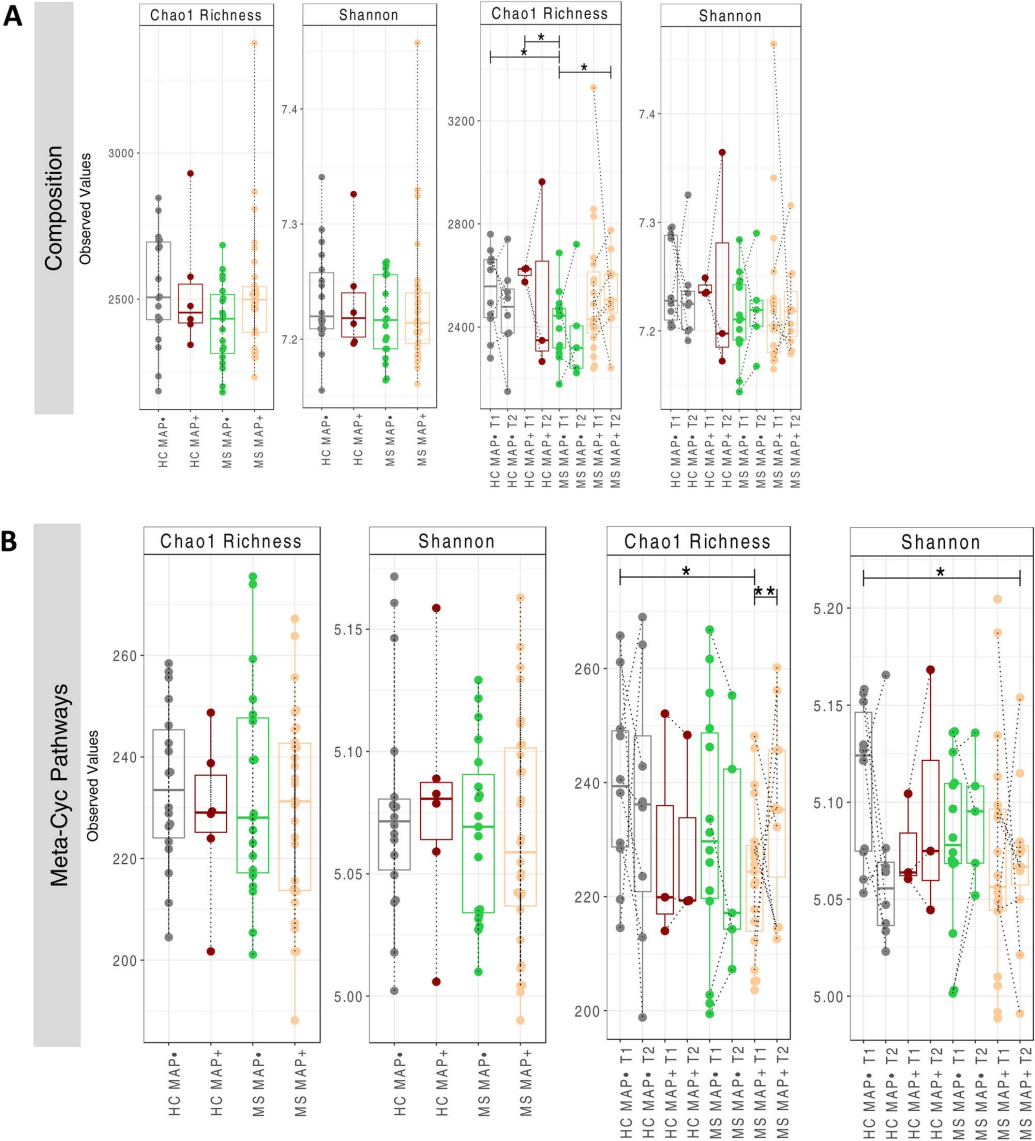
Alpha diversity (Chao 1 Richness and *Shannon* entropy) comparison of (**A**) bacterial OTUs, and the (**B**) MetaCyc pathways predicted from the PICRUSt2 software. Going from left to right, different resolution is considered, i.e., where samples are merged based on time (left panels) and where they are not (right panels). The lines connect samples according to simple ANOVA (left panels) or One-way within ANOVA incorporating paired nature of samples coming from the same subjects (right panels), and where significant are connected with solid lines with significance values as: *p < 0.05, **p < 0.01, or ***p < 0.001. The dotted lines connect two samples when they originate from the same subject.

Microbial alpha diversity is estimated for all study cohorts using Shannon entropy and Chao1 richness as shown in Fig. 1. We calculated these metrics for both the taxonomic abundance table (OTU table) and the functional abundance tables (MetaCyc pathways predicted from the PICRUSt2 software). Samples are classified based on fine granularity with time points T1 and T2 taken separately to coarse granularity where T1 and T2 are merged together for HC MAP−; HC MAP + ; MS MAP−; and MS MAP + . Statistically significant differences (*p* < 0.05) are observed for Chao1 richness between: HC MAP− T1 and MS MAP− T1; HC MAP + T1 and MS MAP− T1; and MS MAP− T1 and MS MAP + T2. Except these, no other statistically significant variation is observed in the alpha diversity among study cohorts. However, considering Meta-Cyc pathways, significant differences (p < 0.05) are observed between: HC MAP− T1 and MS MAP + T1 (Chao1 richness); MS MAP + T1 and MS MAP + T2 (Chao1 richness); and HC MAP− T1 and MS MAP + T2 (Shannon entropy). Note that the above significances incorporate paired nature of subjects using One-way within ANOVA with subject identities.

**Fig. 2:**
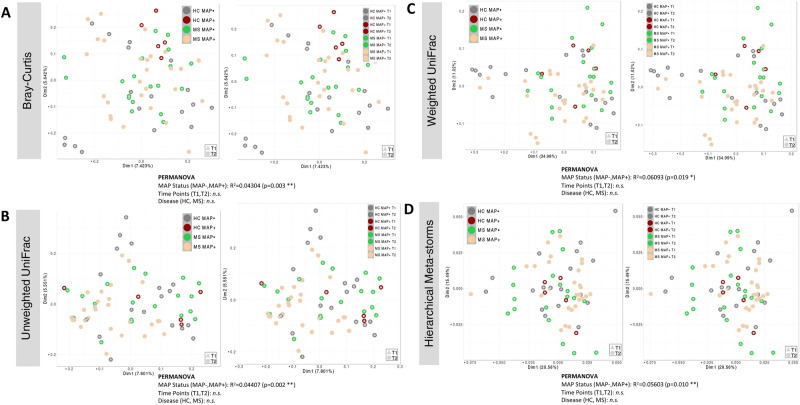
Beta diversity represented by principal coordinate analysis (PCoA) plots with each axis showing the percentage variability explained by that axis, and where ellipses represent 95% confidence interval of the standard error for a given group. We have used four different distance measures: (**A**) Bray–Curtis distance to show differences in composition, (**B**) Unweighted UniFrac distance to show differences in phylogeny, (**C**) Weighted UniFrac to show differences in both composition and phylogeny, (**D**) Hierarchical Meta-storms to show differences in metabolic function. PERMANOVA statistics utilising these distance measures are shown underneath to suggest if there are significant differences between the groups with R^2^ value showing percentage variability explained. Similar to Fig. 1, we have used two different resolutions: merged time points (left panels), and time points taken separately (right panels).

Beta diversity among study groups was also explored using various dissimilarity indices i.e., Bray–Curtis (BC) for compositional variations, Unweighted UniFrac (UWU) for phylogenetic differences, as well as weighted unifrac (WU) for phylogenetic relatedness and Hierarchical Meta-Storms (HMS) for functional analysis (Fig. 2). In human-associated microbiome study, the disease pathology state typically doesn’t have a pronounced shift in community structure as compared to healthy controls, and therefore, in the absence of visual cues in ordination diagrams, a statistical test such as Permutational multivariate analysis of variance (PERMANOVA) is employed to test variability in microbiome composition, phylogeny, and function explained by MAP status (MAP−, MAP +), health status (HC, MS), and time points (T1, T2). For the four beta diversity distances used in PERMANOVA, Bray–Curtis distance (measure of compositional difference), Unweighted UniFrac (measure of phylogeny difference), Weighted UniFrac (measure of abundance weighted phylogeny difference), and Hierarchical Meta-Storms (measure of functional difference) accounted for 4.3%, 4.4%, 6%, and 5.6% variability, respectively. No statistically significant results were observed for health status or time points suggesting MAP infection to be the main driver of change.

### General characteristics of the cohorts and relationship between microbiome and sources of variability

Based on the self-reported questionnaires, the summary statistics are shown in Supplementary Tables S1−S13. Additionally, results from PERMANOVA to associate sources of variability in microbiome structure and function are presented in Supplementary Tables S14, S15. These suggest that MS MAP + individuals experience more sleep restfulness and stomach pain than MS MAP− individuals. Considering all four cohorts (HC MAP-, HC MAP + , MS MAP−, and MS MAP +), using different beta-diversity metrics that account for differences in composition, phylogeny, and function, we have found the MAP status, antibiotics usage, having children and tea consumption without sugar as driver of change in microbiome. PERMANOVA was repeated again on a subset of the samples (MS MAP + , and MS MAP− cohort only) since additional clinical data on medicinal usage was available. We found that the medicinal groups AUBAGIO, CLADRIBINA, COPAXONE, GILENYA, and TECFIDERA alter the microbial composition (using Bray–Curtis distance). Additionally, sex of individuals, disease duration, smoking status, probiotics consumption, work type, antibiotics effect and stool consistency were other main factors that have influenced the change in the microbiome.

### Core microbiota and neutral modelling

**Fig. 3:**
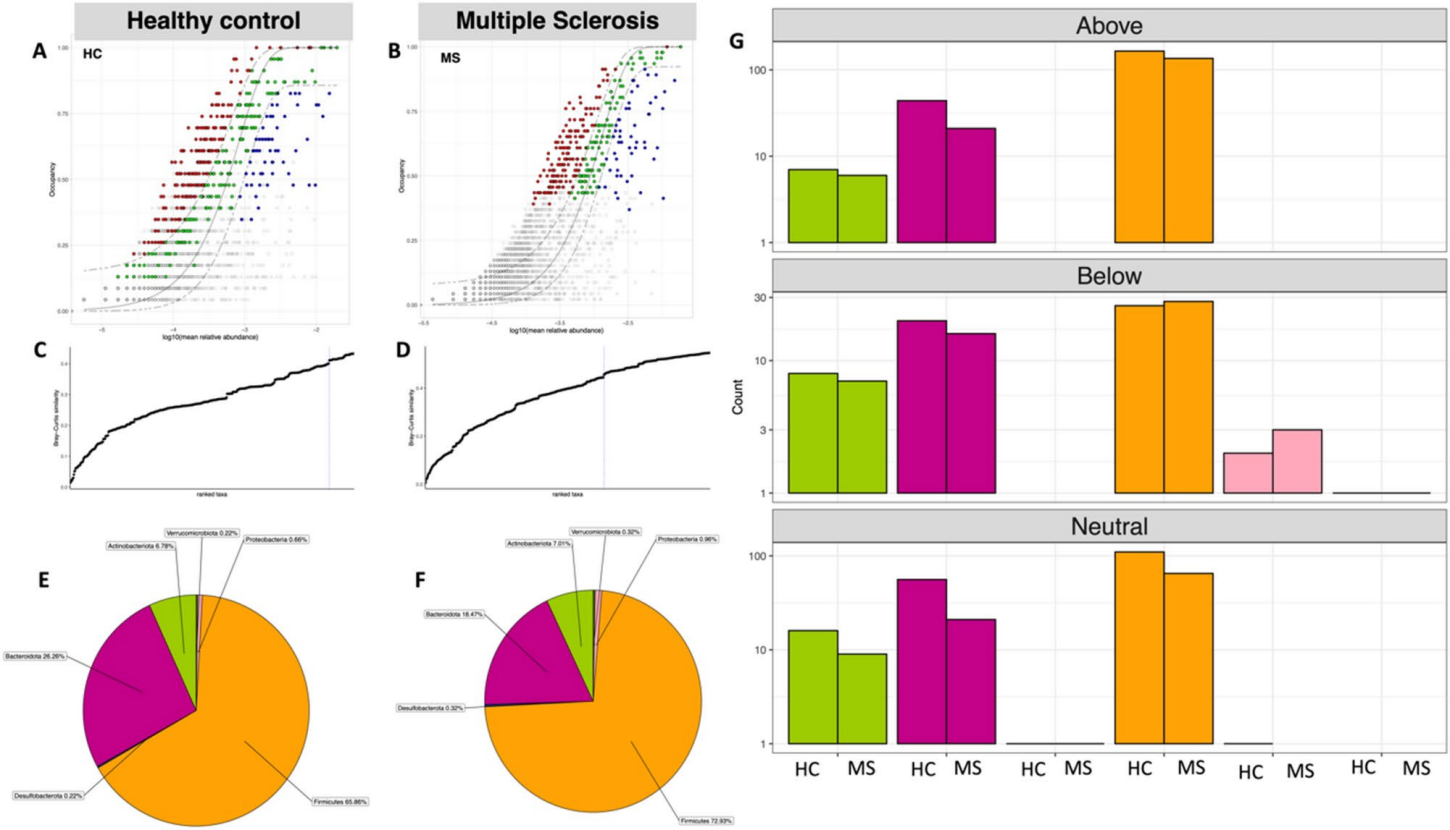
Core microbiome (red, green and blue points) identified through a dynamic strategy for (**A**) HC and (**B**) MS samples. We have used four occupancies in both models (MAP− T1; MAP− T2, MAP+ T1, and MAP+ T2). To identify the thresholds for core microbiome, shown below the abundance-occupancy diagrams for (**C**) HC and (**D**) MS samples, we calculate the function C (that implicitly incorporates explanatory power of the chosen core subset in terms of capturing beta diversity). The dotted lines represent the “last 2% decrease” criteria where OTUs are incorporated in the core subset until there is no more than 2% decrease in beta diversity. Independently, a neutral model is fitted with those OTUs that fall within the 95% interval confidence intervals shown in green, whilst non-neutral OTUs with observed frequency above the predicted frequency from the neutral model (selected by the host) are shown in red colours, and those with observed frequency below the predicted frequency from the neutral model (selected by dispersal limitation) are shown in green colours. The proportion of core OTUs belonging to different phyla are shown with a pie chart for (**E**) HC and (**F**) MS samples whilst the count of neutral/non-neutral OTUs (**G**) are shown with the bar plots.

To explore microbial community assembly whether it is guided by random or deterministic processes, is gaining popularity in recent years. Neutrality is often studied to suggest if the host or the immediate environment is responsible for exerting its influence. To investigate this, we have used a dynamic approach to identify core OTUs from the data, and then coupled it with neutral modelling (Fig. 3). The dynamic approach considers ranking of OTUs (in terms of their membership to core subset) based on a chosen occupancy model (occupancy in both time and MAP infection, i.e. four possible cohorts, MAP− T1, MAP− T2, MAP + T1, MAP + T2 for both HC and MS cohorts). In addition to occupancy of OTUs within these four cohorts, the consistency of OTUs across replicates was also considered to derive the ranking formula (see details of the Sect “Methods” in^24^. With a seed set of highly ranked OTUs as core subset, we inclemently add to this subset OTUs that cause a substantial change in beta diversity by sweeping the ranked OTUs from left to right. We stop including OTUs at a point where the beta diversity increase drop below 2%. Once a final subset of core OTUs are obtained, the neutral model is fitted based on abundance occupancy distributions of these OTUs.

Our results point towards core microbiome (red, green, and blue points) enriched either in diseased or healthy groups. The solid green points depicted the OTUs that fall within the 95% confidence interval and are considered neutral while points above or below this range, are considered deterministic rather than neutral. OTUs having observed frequency greater than the predicted frequency are selected by study groups (i.e., HC and MS) as shown in red color while OTUs that fall below the predicted model frequency are dispersal limited (shown in blue color). We have found a total of 216 and 162 unique core OTUs that were fitted above the 95% confidence interval of the neutral model for HC, and MS cohorts, respectively (Supplementary_Data_Table_S1.csv). These are the ones that are selected by the host environment and are important. Among these, for the MS case, those that were fitted above the neutral model are dominated by [Eubacterium] eligens_group, [Eubacterium] hallii_group, Alistipes, Bacteroides, Bifidobacterium, Blautia, Faecalibacterium, Streptococcus, Subdoligranulum, Dorea, Fusicatenibacter and Roseburia. Among these Blautia, Faecalibacterium, and Bacteroides are the most dominant genus. The healthy core, on the other hand, is enriched with *Bacteroides*, *Bifidobacterium* and *Alistipes* which are mainly commensals depicting the health benefits associated with the presence of these species. Note that the neutral model was fitted based on only one parameter “migration” model and therefore, OTUs that lie below the neutral model are driven by dispersal limitation which implies that the migration is reduced by extraneous factors (host in this case).

The relative abundance of different phyla in the subset of core OTUs is shown by pie charts in Fig. 3 for both HC and MS. The core microbial communities are mainly dominated by *Firmicutes* and *Bacteroidetes* in both HC and MS groups, whilst *Actinobacteria*, *Desulfobacterota*, *Proteobacteria*, and *Verrucomicrobiota* are the least abundant phyla. Although the core microbiome analyses returned the same phyla for the HC and MS cohort, the number of OTUs belonging to these phyla differed. Based on neutrality analysis, majority of the OTUs influenced by the host were *Firmicutes*.

The taxonomic coverage of the core OTUs at different occupancy (MAP− T1, MAP− T2, MAP + T1, and MAP + T2) is shown in Supplementary Figures S2,S3 for both HC and MS groups. At T1, the clade of *Eggerthellaceae* was abundant in HC MAP + T1 in comparison to HC MAP + T2. However, at T2, clade of *Acidaminococcales* and *Verrucomicrobiota* exhibited high abundance in HC MAP + T2 in comparison to MAP + HC T1. There were no significant changes in HC MAP- between both time points. In MS cohort, *Prevotellaceae* and *Ruminococcus* were abundant at T2 in MS MAP−. *Coriobacteriales* was more abundant in MS MAP + T2 and *Negativicutes* were less abundant in T2 as compared to T1.

### Differential abundant taxa analysis

**Fig. 4:**
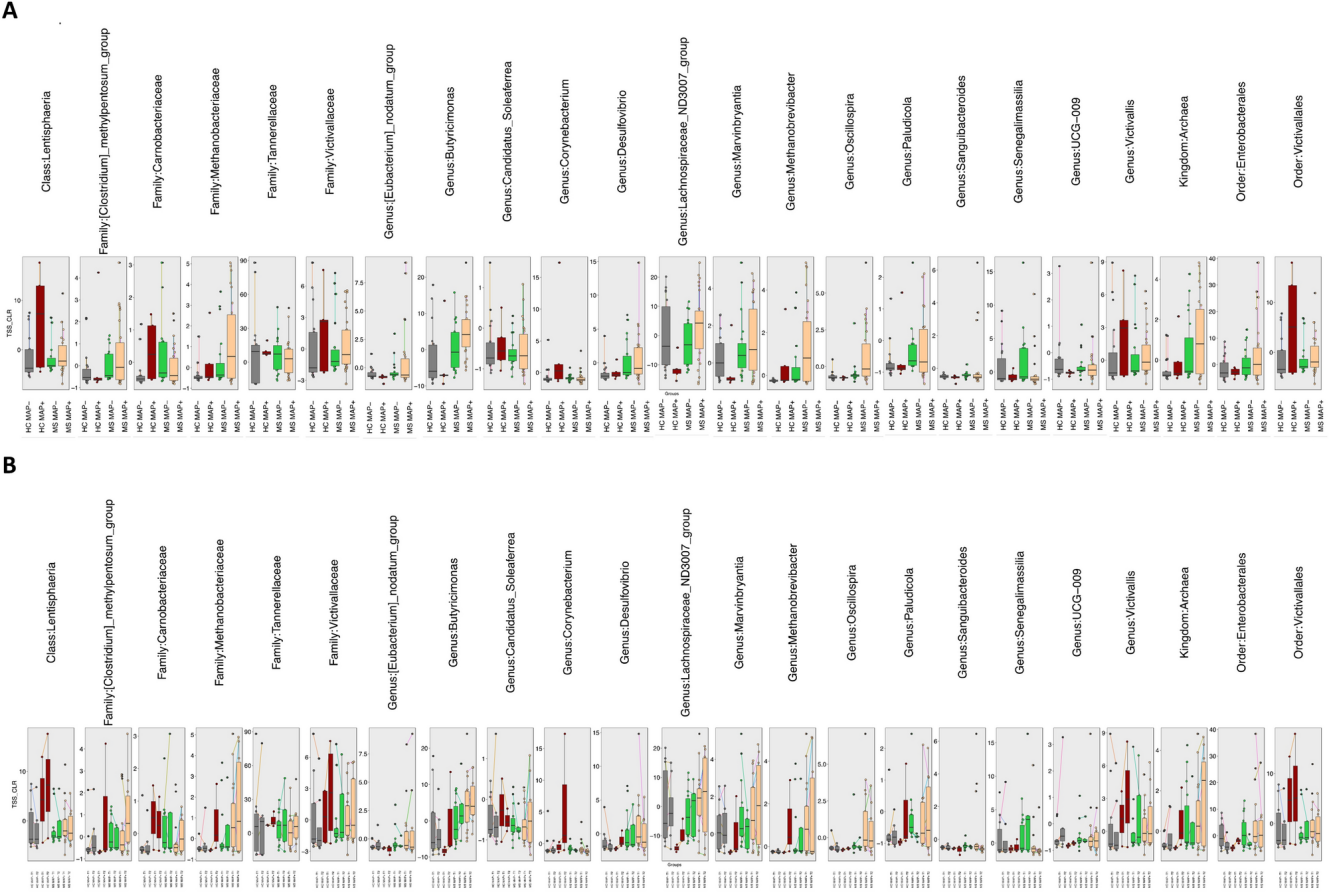
Subset of taxa (at different lineages, *Kingdom, Phylum, Class, Order, Family, Genus*) returned from QCAT-C association test that are differentially abundant between the cohorts considered in this study, where (**A**) merges samples from time points T1 and T2 together, and (**B**) considers them separately. The QCAT-C association test that takes into account paired nature of samples i.e., originating from the same subject, and are connected by lines. The values represent the TSS+ CLR normalized abundances of individual taxa. The global P-value is the test associated with the collective subset returned as significantly different, whilst the local P-values < 0.05 (not shown here) for all features.

**Fig. 5:**
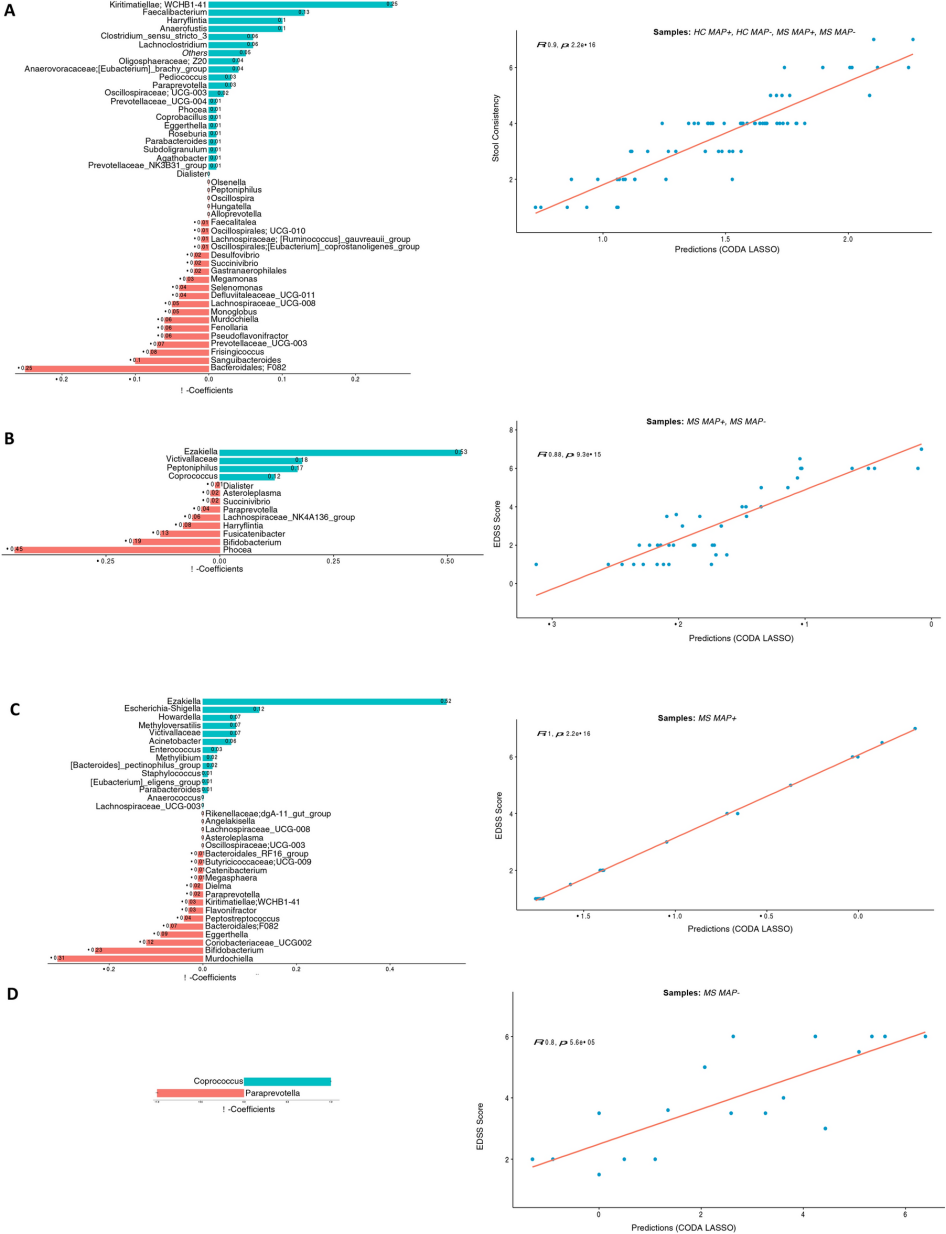
Two disjoint sets of $\beta -$ coefficients for OTUs collated at genus level, with those that are positively associated are shown in green, whilst those that are negatively associated are shown in red. These are returned from apply the CODA-LASSO procedure using the following outcomes: (**A**) *Stool Consistency* (for HC, MAP + , and MAP − samples) (**B**) *EDSS Score* (for MAP + , and MAP − samples) (**C**) *EDSS Score* (for MAP + samples only), and (**D**) *EDSS Score* (for MAP − samples only). The prediction accuracy with R value as a quality of fit criteria, is shown on the right side and shows good agreement between the prediction obtained through the models and the true values.

Due to the paired nature of data, with multiple subjects providing two samples points at time point T1 and T2, respectively, we have used a specialized QCAT-C association test that reduces Type 1 errors due to correlations introduced by paired-nature and gives differential taxa at different lineages. The differential abundant taxa at genus, family, order, class, and phylum level are shown in Fig. 4. Irrespective of the granularity considered, we have found distinct microbial community profile in MS patients as compared to the HC individuals that include: kingdom Archaea; families *[Clostridium]_methypentosum_group,* and *Methanobacteriaceae*; order *Enterobacterales* as well as Genera *Methanobrevibacter*, *Marvinbryantia*, *Lachnospiraceae_ND3007_group*, *Butyricimonas*, *Paludicola*, *[Eubacterium]_nodatum_group*, *Desulfovibrio* and *Oscillospira* (Fig. 5). At coarser granularity (HC MAP − ; HC MAP + ; MS MAP − ; and MS MAP +), the above-mentioned microbial species were observed in higher abundance in MAP + individuals at different taxon level. This give credence to the hypothesis that greater gut dysbiosis is observed when MS patients are infected with various gut infection i.e., MAP infection in our case.

### Continuous covariates and their association with the key microbes and pathways via CODA-LASSO regression

**Fig. 6:**
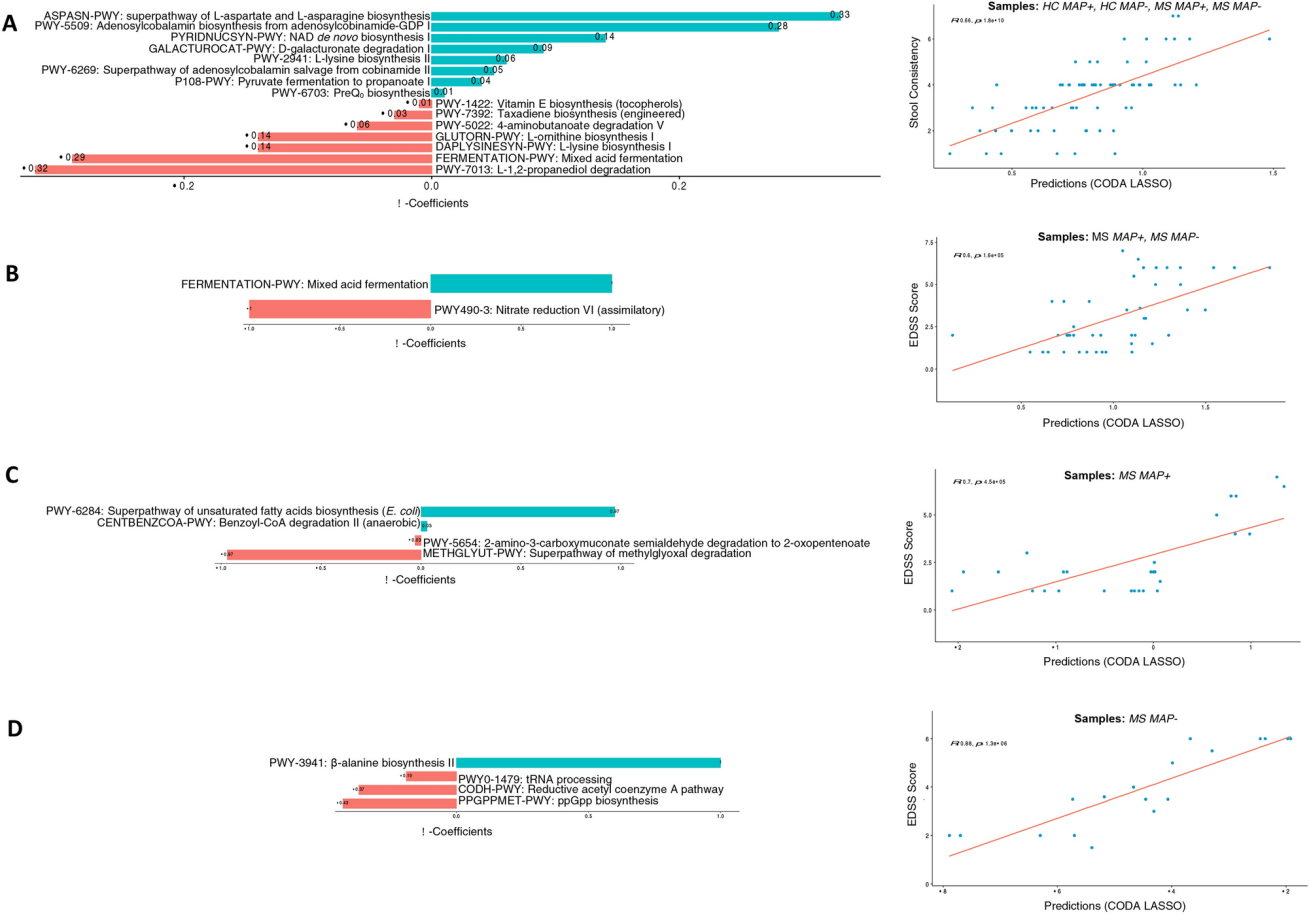
Two disjoint sets of $\beta -$ coefficients for MetaCyc pathways, with those that are positively associated are shown in green, whilst those that are negatively associated are shown in red. These are returned from apply the CODA-LASSO procedure using the following outcomes: (**A**) *Stool Consistency* (for HC, MAP + , and MAP − samples) (**B**) *EDSS Score* (for MAP + , and MAP − samples) (**C**) *EDSS Score* (for MAP + samples only), and (**D**) *EDSS Score* (for MAP − samples only). The prediction accuracy with R value as a quality of fit criteria, is shown on the right side and shows good agreement between the prediction obtained through the models and the true values.

**Fig. 7:**
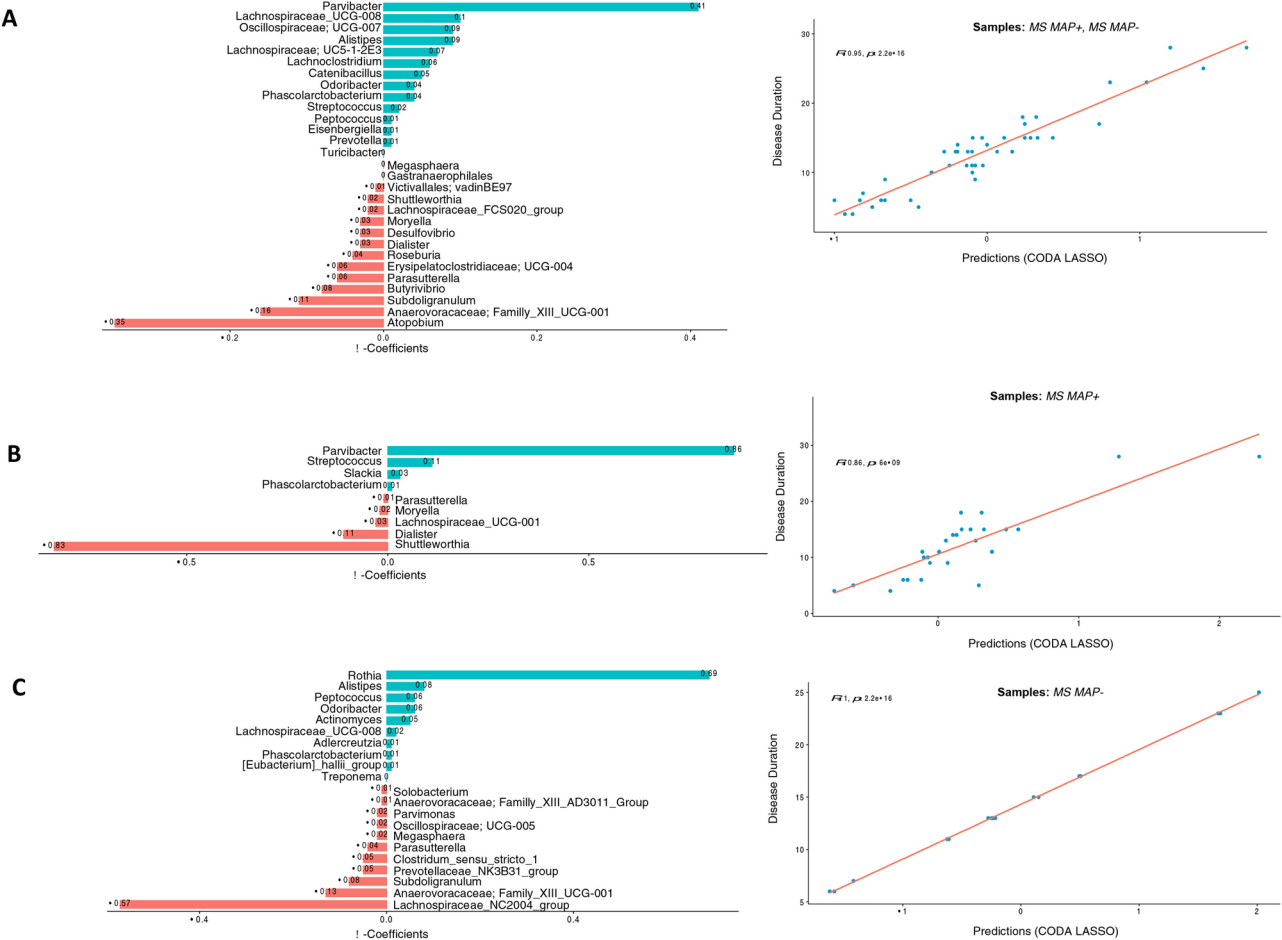
Two disjoint sets of $\beta -$ coefficients for OTUs collated at genus level, with those that are positively associated are shown in green, whilst those that are negatively associated are shown in red. These are returned from apply the CODA-LASSO procedure using the following outcomes: (**A**) *Disease Duration* (for MAP + , and MAP − samples) (**B**) *Disease Duration* (for MAP + samples only), and (**C**) *Disease Duration* (for MAP − samples only). The prediction accuracy with R value as a quality of fit criteria, is shown on the right side and shows good agreement between the prediction obtained through the models and the true values.

**Fig. 8:**
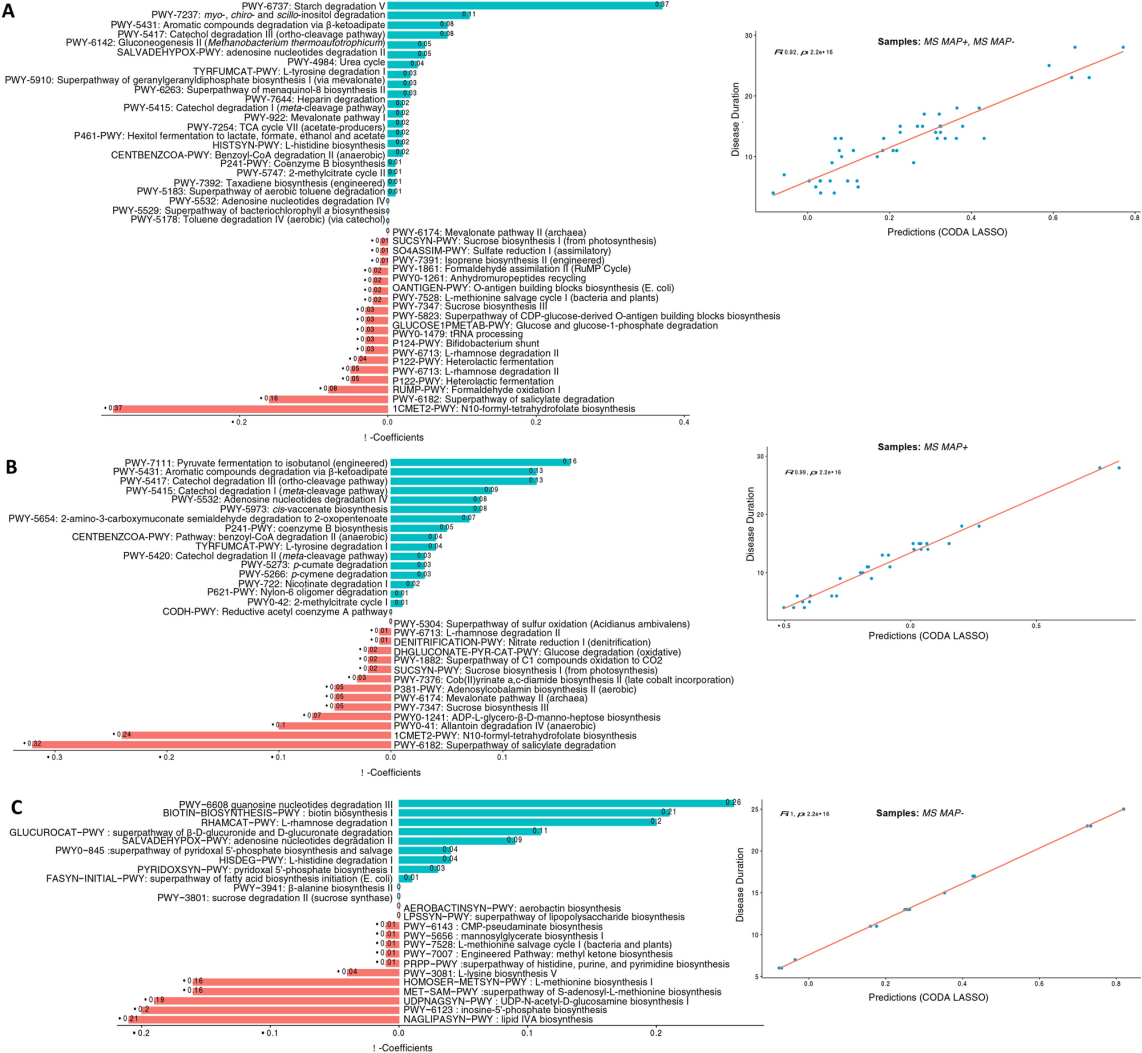
Two disjoint sets of $\beta -$ coefficients for MetaCyc pathways, with those that are positively associated are shown in green, whilst those that are negatively associated are shown in red. These are returned from apply the CODA-LASSO procedure using the following outcomes: (**A**) *Disease Duration* (for MAP + , and MAP − samples) (**B**) *Disease Duration* (for MAP + samples only), and (**C**) *Disease Duration* (for MAP − samples only). The prediction accuracy with R value as a quality of fit criteria, is shown on the right side and shows good agreement between the prediction obtained through the models and the true values.

In the current study, we have also recorded continuous covariates such as *Stool consistency*, *EDSS score*, and *Disease duration*. To find the relationship between these parameters and the subset of microbes or pathways that change when the above covariates vary, we employ a variable selection approach called CODA LASSO Regression. The regression returns two distinct subsets of features (microbes or pathways), with those that increase (with positive beta coefficient), and those that decrease (with negative beta coefficient) against the covariate of interest. Whilst using this approach, it is important which samples to use in the model as some of the data (EDSS score, and Disease duration) are only available for MS individuals (MAP− and MAP + included), and therefore, we have not included HC samples in the analyses (case 2). On the other hand, *Stool consistency* is available for all samples including HC, and therefore, CODA LASSO is run on all samples (case 1). For MS MAP + (Case 3) and MS MAP− (case 4) individuals, we also ran the regression model separately using *EDSS score*, and *Disease duration*. The results for applying these regression models for composition and pathways tables are shown in the Figs. 5, 6, 7, 8.

When considering case 1 (HC MAP-, HC MAP + , MS MAP-, and MS MAP +) and using *Stool consistency*, we found a positive association of key bacterial commensals, e.g., *Kiritimatiellae; WCHB1-41*, and *Faecalibacterium* whilst *Bacteroidales F082* displayed a negative association with the *Stool Consistency* (Fig. 5A). On the other hand, to determine which bacterial species are linked with *EDSS score*, we examined its relationship for case 2 (MS MAP− and MS MAP + patients), case 3 (MS MAP +) and case 4 (MS MAP−), respectively. *Ezakiella* was positively associated with the *EDSS score* in cases 2 and 3, whilst *Coprococcus* was positively associated in cases 2 and 4, respectively. In terms of *EDSS score Phocea*, *Murdochiella* and *Paraprevotella* species have shown a negative correlation with above mentioned cases (Fig. 5B–D).

The functional disparity among different groups was also explored using MetaCyc pathways. Considering *stool consistency* and *EDSS score* with all above mentioned cases, we found a positive association of amino acids and fatty acids biosynthesis pathways i.e., superpathway of l-aspartate and l-asparagine biosynthesis; mixed acid fermentation; superpathway of unsaturated fatty acids biosynthesis (*E. coli*); and β-alanine biosynthesis II (Fig. 6). However, certain degradation and reduction pathways, are found to be negatively associated with all cases i.e., l-1,2-propanediol degradation; nitrate reduction VI (assimilatory); superpathway of methylglyoxal degradation; and reductive acetyl coenzyme A pathway. We also explored the relationship of key microbial species (Fig. 7) and MetaCyc pathways (Fig. 8) with the *Disease status*. The results have shown a significant positive association of *Parvibacter* in cases 2 and 3 (that both contain MS MAP + individuals) while *Rothia* was significantly positively associated in case 4 (MS MAP− individuals). *Atopobium*, *Shuttleworthia* and *Lachnospiraceae_NC2004_*group have typically shown a negative association with the *Disease Status*. We also found positive association of starch degradation V and guanosine nucleotides degradation III in case 2 and 4 (that both contain MS MAP− individuals) while fermentation pathways are significantly positively associated in case 3 (MS MAP + individuals only).

### Key microbial community members contributing to beta diversity differences across samples using BVSTEP routine

Through a permutation approach called BVSTEP routine (results shown in Supplementary Figure S4), we are able to deduce 19 OTUs from the top 4000 most abundant OTUs, that roughly conserve the same beta diversity distance (Bray–Curtis) between samples (a correlation of R = 0.82850) as the full set of 20,634 OTUs. These resulting highly variable subsets of OTUs belonged to the genera *Faecalibacterium*, *Bacteroides*, *Lachnospiraceae*, *Alistipes*, *Agathobacter*, *Ruminococcaceae*, *Prevotella*, and *Fusicatenibacter*. Notably, OTU_1 (*Faecalibacterium*), OTU_10 (*Bacteroides*), OTU_100 (*Bacteroides*), and OTU_1005 (*Lachnospiraceae*) were found to be the key OTUs that vary significantly between the study groups (HC MAP−, HC MAP + , MS MAP−, and MS MAP +). OTU_100 and OTU_1005 were mainly dominant in the MS MAP + individuals as compared to the MS MAP− and HC cohort (HC MAP−, HC MAP +). In contrast, OTU_10 were abundant in HC individuals.

### Mediation analysis, and role of microbes as mediators

So far, the reported analyses concentrated on bivariate associations whether, certain microbes are up-/down-regulated with respect to a categorical outcome (treatment group) or increasing/decreasing with respect to a continuous outcome (clinical parameters). To unravel the complex interplay between different sources of variability including anthropometric and sociodemographic data deemed as confounders, and different treatment groups (MS MAP + , MS MAP −) and outcome (disease duration), we have identified microbes that play a mediatory role. To be a mediating microbe, the requirement is for a tripartite relationship (*MAP Status*—*Microbe—Disease Duration*) to exist. These microbes are highlighted in Supplementary Table S16 to have two significant Q-values (< 0.05) simultaneously, one for *MAP Status—Microbe* link, and one for the *Microbe—Disease Duration* link. In addition, based on several global tests, we have identified sufficient number of microbes to have a global mediation effect.

**Fig. 9:**
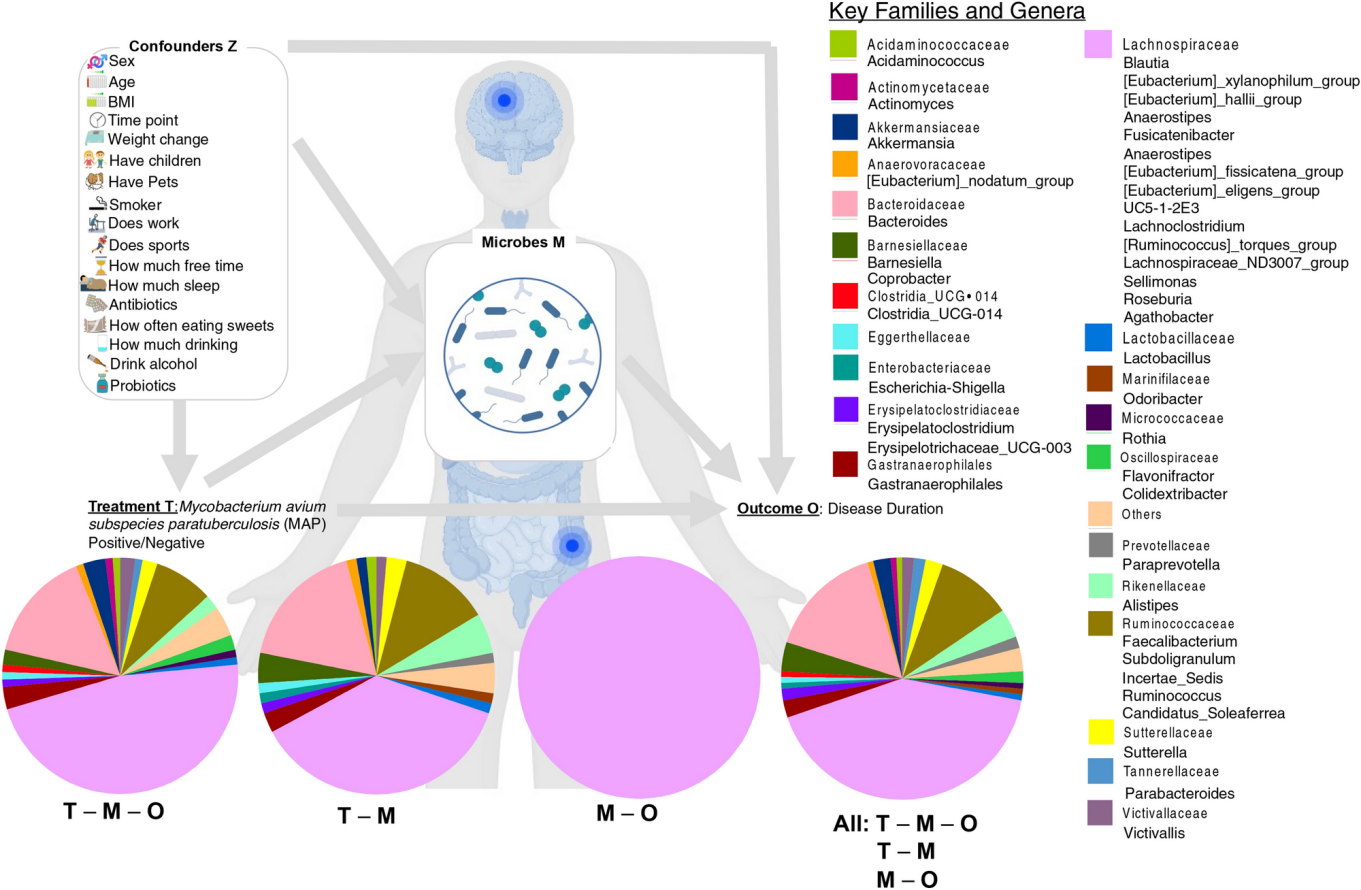
Summary statistics of OTUs playing a mediation role between T (MAP − /MAP +) and outcome (disease duration), and coloured according to their taxonomic assignment at Family level, and based on Supplementary Table S16 . The four pie charts represent: *T – M – O tripartite relationship* with T – M Q-value ≤ 0.05 and M–O Q-value ≤ 0.05; *T-M bivariate relationship* with T – M Q-value ≤ 0.05 and M–O Q-value > 0.05; *M–O bivariate relationship* with T – M Q-value > 0.05 and M–O Q-value ≤ 0.05; and *all relationship* with T – M Q-value ≤ 0.05 or M–O Q-value ≤ 0.05. The pie chart then represents the proportional representation of families within these types of relationship. The model also takes into account confounders Z shown on top left.

The summary statistics of main microbial mediators are shown in Fig. 9. Majority of these OTUs where tripartite relationship (T–M–O) exists, were resolved at species level, and belonged to *Sutterella wadsworthensis*, *Schaalia odontolytica*, *Acinetobacter sp.*, *Alistipes finegoldii*, *Clostridium leptum*, *Ruminococcus bicirculans*, *Azospirillum sp.*, *Victivallis vadensi*, and *Blautia hydrogenotrophica*.

## Discussion

The existing literature corroborates the association of multiple sclerosis (MS) with inflammatory bowel disease (IBD)^25,26^. As compared to the general population, MS patients are at a high risk of developing IBDs^27,28^. Also noteworthy is the increased prevalence of demyelinating diseases in IBD patients^26^. Additionally, genome wide association studies considering Crohn’s disease and Ulcerative colitis have revealed a shared risk locus between IBD and MS indicating a common underlying pathological mechanism affecting both conditions^29^. Therefore, one would expect the microbial signature between MS and IBD to be similar. With a rich body of supporting evidence, we have therefore designed this study to explore the gut microbiota in view of the effect of *Mycobacterium avium subspecies paratuberculosis* (MAP) in MS patients, which is also implicated in Crohn’s disease^30–32^.

To our knowledge, this is the first study to highlight the differences in the gut microbiome of MS patients with MAP infection (MAP +) compared to those without MAP (MAP-), using healthy controls (HCs) as a baseline. The strength of our study lies in the utility of several advanced analytical tools including differential abundance, core microbiome and neutral modelling analyses, which provide an unprecedented level of control on discriminatory patterns that may have biological relevance. Moreover, mediation analysis has added further credence by identifying the microbes that play a mediatory role between variations in the treatment groups (MS MAP + /MS MAP−), confounders, and the disease duration as an outcome variable.

In this study, we have not found any significant differences in terms of alpha diversity between the study cohorts (HC MAP−, HC MAP + , MS MAP−, and MS MAP +) in both microbial composition and their functional potential without considering the time points supporting the previous literature^33,34^. Whilst there were very few significant results, the diseased groups (MS, MAP + , MAP−) exhibited slightly lower diversity than the HCs indicating that reduction in gut diversity might be a reason to exacerbate the disease^35^. In terms of beta diversity, there was from 4 to 6% variability in the studied cohort when considering microbial composition, their phylogeny and function. Based on PERMANOVA, the major source of variation was MAP status (4–6% variability in different beta-diversity metrics) and not time (T1, T2) nor disease (HC, MS) suggesting MAP infection to play a major role.

Typically, the core microbial species exhibit functional redundancy that is expected to stabilize the ecosystem, and also contain specialized functions that have the potential to shape the microbiome landscape. The definition of core microbiome is debatable, and often implies a crisp threshold to decide which part of microbiota is prevalent in majority of the samples^36^. To circumvent this, we have used a recently published dynamic approach where core membership is learnt based on the explanatory power of core subset in a beta diversity space^37^. Furthermore, by combing with the neutral modelling, we are able to ascertain the influence of host environment in shaping the core communities. Irrespective of the group considered, majority of the core species selected by the host belonged to the *Firmicutes* phyla.

Differential abundance analyses have revealed that majority of the species that become dominant in MS MAP + individuals, such as *Methanobrevibacter*, *Marvinbryantia*, *Butyicimonas* and *Lacchnospiracea-ND307-group*, are also linked with higher inflammation rate and considered to be the signature of gut microbial dysbiosis in MS ^38–41^. On the other hand, the dominant microbiota in HC individuals comprise of class *Lentisphaeria* and family *Victivallaceae* that are often the indicator species of a healthy microbiota^42,43^. Using CODA-LASSO regression, we have identified genera that are either positively or negatively associated with *Stool consistency*, *Expanded Disability Status Scale* (*EDSS) score*, and *Disease duration*. *Ezakiella* (positively associated with EDSS) implicated in in MS MAP + and MS MAP− individuals is in line with the previously published literature where *Ezakiella* was declared as a potential predictive biomarker of RRMS^44^. At the same time, SCFAs producing *Coprococcu*s is also found to be depleted which corroborates previous studies^45–47^. In case of *Disease duration,* we found *Parvibacter* as significantly positive association in MS MAP + individuals whilst *Rothia* being more positively associated with MS MAP− individuals, also reported in^48^. Other pronounced association of microbiota with *Stool consistency* through CODA-LASSO procedure including above identify genera that could be potentially used as biomarkers in future intervention studies. CODA-LASSO regression also revealed association of above covariates with MetaCyc pathways. The significantly positively associated pathways in all cases (using *EDSS score* and *Stool consistency*) belong to biosynthesis pathways of amino and fatty acids i.e., superpathway of l-aspartate and l-asparagine biosynthesis, mixed acid fermentation, superpathway of unsaturated fatty acids biosynthesis (*E. coli*) and β-alanine biosynthesis II which may all be required for energy production, cellular growth and essential molecule synthesis. We also found positive association of complex sugar degradation pathways (starch degradation V, guanosine nucleotides degradation III) in MS cohort, particularly for MS MAP − group, whilst fermentation pathways (pyruvate fermentation to isobutanol) are dominant for MS MAP + individuals. BVSTEP routine identified major beta diversity changes between all samples due to Bacteroides. It is abundant in both HC and MAP + individuals due to its nature as opportunistic bacteria that implies both beneficial as well as pathogenic roles in humans^49^.

Our findings from mediation analysis also revealed that *Acinetobacter* and *Akkermansia* are the main microbial species that have a mediatory role. These species act as inflammatory marker^50,51^ having molecular mimicry with brain antigens^52^ and are also involved in MS pathology^38,53,54^.

Overall, our findings revealed that the dominant genera, *Methanobrevibacter*, *Lachnospiraceae*, and at Kingdom level, *Archaea*, were altered in the entire MS cohort (MS MAP + and MS MAP− included). This suggests that the changes are not specifically linked to MAP infection. However, within MS MAP + individuals, we found abundance of certain species such as *Prevotellaceae*, *Escherichia-shigella*, *Desulfovibrionaceae* and *Ezakiella*, that have diagnostic capability to discriminate between MS MAP + and MS MAP− individuals. Multiple species of *Escherichia-shigella* are known to be either pathogenic or opportunistic^55,56^ having correlation with intestinal inflammation markers i.e., C-reactive proteins (CRP). Pathogenic species may stimulate inflammation by suppressing inflammatory or epithelial cell autophagy^57^. *Ezakiella* species are normally associated with the consumption of high carbohydrate diet^58,59^. Navarro-López et al. (2022) found the association of *Ezakiella* with relapse remitting multiple sclerosis (RRMS) and declared it as a risk factor in RRMS patients^44^. Numerous studies have found that *Desulfovibrionaceae* are more common in IBD patients than in healthy individuals. It is known that MAP is one of the causes of IBD. At the same time higher abundance of *Desulfovibrionaceae* was also found in our MAP + cohort, suggesting a link between MAP infection and the higher abundance of these bacteria^60–62^. With preponderance of evidence, our findings confirm the role of MAP infection in gut microbiota of patients with multiple sclerosis. Furthermore, the microbial dysbiosis is not only linked with the disease status but also with gut infections that stimulate various inflammatory associated species that either activate autoimmunity or inflammation related pathways and worsen the disease condition.

Though our study gives preliminary (with small sample size) albeit comprehensive insights into the potential involvement of MAP infection in MS and gut dysbiosis, there are certain limitation that need to be addressed in the future studies. The first consideration is the potential influence of study specific confounders (i.e., antibiotics, pregnancy, medication taken before or after, dietary intake etc.) on our research findings. To mitigate the effect of these confounders, we must either set a stringent exclusion criterion that rules out the effect of these factors on gut microbial composition or employ an analytical procedure (such as mediation analysis in our case) which marginalises for the effect of confounders. Whilst previous studies have established a link between key confounders such as age, gender, and BMI with changes in the bacterial composition^63,64^, because of the narrow range of inclusion criteria in our study, we are unable to identify significant microbial community changes. Future studies that consider a wide range of socio anthropometric measurements is desirable. As for other clinical parameters, we also observed a narrow range of their values, for example, majority of the patients have reported EDSS score below 4 (MS MAP + : median = 2; and MS MAP−: median = 3.55). As a result, whilst PERMANOVA was unable to associate global changes in microbiota with EDSS scores, subtle changes were recovered through CODA-LASSO regression.

A potential limitation of this work is that the metabolic results are based on metabolic profiles predicted through PICRUSt2, which have not been confirmed through quantitative PCR or other measures. Shotgun metagenomics of these samples would more accurately identify the functional changes. Nonetheless, PICRUSt2 has been shown to perform well on human-associated microbiome datasets. This is mainly due to a comprehensive reference database of genomes whose functions are already known, with a tenfold increase in the numbers since the previous release, and as a result, increase our confidences in the pathways found to be differential^23^. The study could also benefit from inclusion of other modalities such as metabolomics, transcriptomics of host species.

Our work elucidates the microbial ecology of individuals diagnosed with multiple sclerosis when they have Mycobacterium avium subspecies paratuberculosis (MAP) infection, a largely unexplored topic in scientific literature. We have employed advanced bioinformatics tools to unravel the complex interplay between microbiome, exposome, and other clinical parameters. We have used a dynamic core microbiome analysis that incorporates temporal occupancy model and neutral modelling to identify the signature microbiome of MAP infection and have additionally identified microbes that play a mediating role. Having characterised the key microbes associated with different sources of variation, and disease status, our study may play a vital role in designing personalised dietary and lifestyle intervention to manage the microbes as well as MAP infection.

## Electronic supplementary material

Below is the link to the electronic supplementary material.

Supplementary Materials

Supplementary Table S1.

Supplementary Table S2.

## Data Availability

The raw sequence files supporting the results of this article are available in the European Nucleotide Archive under the project accession number PRJEB67783 with details of the samples provided in Supplementary_Data_Table_S2.csv. Sequence data that support the findings of this study have been deposited in the European Nucleotide Archive with the primary accession code PRJEB67783.

## Abbreviations

16SrRNA: 16S ribosomal RNA
MS: Multiple sclerosis
RRMS: Relapse remitting multiple sclerosis
BMI: Body mass index
MALT: Mucosa-associated lymphoid tissue
T1D: Type 1 diabetes
IBD: Inflammatory bowel disease
CD: Crohn’s disease
PD: Parkinson’s disease
MAP: *Mycobacterium avium subspecies paratuberculosis*
OTUs: Operational taxonomic units
BC: Bray–Curtis
UWU: Unweighted UniFrac
WU: Weighted unifrac
HMS: Hierarchical meta-storms
PERMANOVA: Permutational multivariate analysis of variance
PCoA: Principal coordinate analysis
QCAT-C: Quasi conditional association test-cluster
*EDSS*: Expanded disability status scale
