# Supplementary material for: Mycobacterium avium subspecies paratuberculosis (MAP) infection, and its impact on gut microbiome of individuals with multiple sclerosis
Source: Sci Rep. 2024 Oct 14;14:24027. doi: 10.1038/s41598-024-74975-4 (PMC11479286; doi:10.1038/s41598-024-74975-4)
Supplement: Supplementary file 1 — Supplementary Materials [file 41598_2024_74975_MOESM1_ESM.docx]

**Supplementary Materials**

***Mycobacterium avium subspecies paratuberculosis* (MAP) infection, and its impact on gut microbiome of individuals with Multiple Sclerosis**

Hajra Ashraf^1,4^, Plamena Dikarlo^2^, Aurora Masia^3^, Ignazio R. Zarbo^3^, Paolo Solla^3^, Umer Zeeshan Ijaz^4,5,6,*^, Leonardo A. Sechi^1,7,*^

^1^Department of Biomedical Sciences; University of Sassari, Sassari, Italy

^2^BIOMES NGS GmbH, Schwartzkopffstraße 1, 15745, Halle 21, Wildau, Germany

^3^Department of Medicine and Pharmacy, Neurology, University of Sassari, Italy

^4^Water & Environment Research Group, University of Glasgow, Mazumdar-Shaw Advanced Research Centre, Glasgow, United Kingdom

^5^National University of Ireland, Galway, University Road, Galway, Ireland

^6^Department of Molecular and Clinical Cancer Medicine, University of Liverpool, Liverpool, United Kingdom

^7^ Complex Structure of Microbiology and Virology; AOU Sassari, Sassari, Italy

* **Joint corresponding authors** (both authors jointly directed this work)

**Contact:**

Hajra Ashraf, [h.ashraf@studenti.uniss.it](mailto:h.ashraf@studenti.uniss.it)

Plamena Dikarlo, [plamena.dikarlo@biomes.world](mailto:plamena.dikarlo@biomes.world)

Aurora Masia, [masia.aurora@tiscali.it](mailto:masia.aurora@tiscali.it)

Ignazio R. Zarbo, [irzarbo@uniss.it](mailto:irzarbo@uniss.it)

Paolo Solla, [psolla@uniss.it](mailto:psolla@uniss.it)

Umer Zeeshan Ijaz*, [Umer.Ijaz@glasgow.ac.uk](mailto:Umer.Ijaz@glasgow.ac.uk) (<http://userweb.eng.gla.ac.uk/umer.ijaz>)

Leonardo A. Sechi*, [sechila@uniss.it](mailto:sechila@uniss.it)

**Statistical Analysis**

As a pre-processing step, we selected for samples with >5000 reads, removed typical contaminants such as *Mitochondria* and *Chloroplasts*, as well as any Operational Taxonomic Units (OTUs) that were unassigned at all levels, as per recommendations given at <https://docs.qiime2.org/2022.8/tutorials/filtering/> giving a final table of n=69 x P=16,787 OTUs with the summary statistics of reads mapping to these OTUs for samples as follows: [Minimum: 5,074; 1^st^ Quartile: 14,380; Median: 18,060; Mean: 20,634; 3^rd^ Quartile: 22,651; Maximum: 96,572].

The ’s vegan package (1) was used for alpha and beta diversity analyses. For alpha diversity measures we have used (after rarefying to minimum library size): (i) *Shannon entropy* – a commonly used index to measure balance within a community; (ii) *Chao1 richness* – the estimated number of species/features in a rarefied sample. We have used R's aov() function to calculate the pair-wise analysis of variance (ANOVA) p-values which were then drawn on top of alpha diversity figures. To adjust for the paired-nature of the samples, i.e. coming from the same subjects, all the pairwise statistics were done using One-way within ANOVA (<http://www.cookbook-r.com/Statistical_analysis/ANOVA/>) as aov(value ~ Groups + Error(SubjectID/Groups)) with samples coming from the same subjects connected with lines. Here, the “Groups” are HC MAP- T1, HC MAP- T2, HC MAP+ T1, HC MAP+ T2, MS MAP- T1, MS MAP- T2, MS MAP+ T1, and MS MAP+ T2, whilst “value” represent the rarefied Chao1 richness or Shannon entropy values.

To visualise the abundance table, we have used Principal Coordinate Analysis (PCoA) with different distance measures. Specifically, we have used three different measures in PCoA: (i) *Bray-Curtis distance* on the OTU abundance table to visualise the compositional changes; (ii) *Unweighted UniFrac distance* estimated using R’s Phyloseq package (2) to see changes between samples in terms of phylogeny; and (iii) *Hierarchical Meta-Storms* (HMS) (3), a recent functional beta diversity distance which takes the observed KEGG Orthologs (KOs) recovered from the dataset, and then calculates the functional beta diversity distance in a hierarchical fashion propagating the KOs abundances upward to the pathways in a multi-level pathway hierarchy to give a weighted dissimilarity measure. Additionally, Vegan package was also used to perform PERMANOVA analyses to see if the microbial or functional community structures can be explained by different sources of variability. In addition to the above-mentioned distance measures, we have also employed weighted UniFrac distances in the PERMANOVA by using the Phyloseq package.

Since the data for subjects are paired, i.e., same individuals provided multiple samples at time point T1 and T2, we have also used a specialised cluster association test (4) utilising R’s miLineage package (<https://tangzheng1.github.io/tanglab/software.html>) with this test referred to as QCAT-C test using the QCAT_GEE. Cluster () function (with default values) from the package. The test is robust to deduce complex correlations that exist among microbes due to paired nature of samples. Additionally, the QCAT-C test is a two-part test where it fits separate models to microbes that are excessively zero, and those that are not, referred to as positive microbes, based on the taxonomic tree to localize the covariate-associated lineages. As a result, the differential abundance analysis of microbes gives better estimates and reduces Type 1 errors. To visualise the differentially abundant taxa at different taxonomic ranks, we have used *Total Sum Scaling* followed by a *Centralized Log Ratio* (TSS+CLR) transformation on the raw abundance values.

To find a minimal subset of genera that changed between quantitative outcome (stool consistency, and EDSS score), we have used the CODA LASSO (5) of the form $y_{i}=\beta_{0}+\beta_{1}\log\left( x_{1i} \right)+\ldots+\beta_{j}\log\left( x_{ji} \right)+\epsilon_{i}$ (for $i$-th sample and $j$-th microbe, with $x_{ji}$ being the abundance of genera), and where the outcome $y_{i}$ is a binary outcome variable (uses logistic regression). The model uses two constraints: a) $\sum_{k\geq1} \beta_{k}=0$ (i.e., all $\beta$-coefficients sum up to 1) which makes the algorithm invariant by returning two disjoint sets of features in a log contrast fashion; and b) the optimization function incorporates a LASSO shrinkage term $\lambda\sum_{k\geq1} \left| \beta_{k} \right|$ as $\sum_{i=1}^{n} (y_{i}-\beta_{0}-\beta_{1}\log\left( x_{1i} \right)-\ldots-\beta_{j}\log{\left( x_{ji} \right))}^{2}+\lambda\sum_{k\geq1} \left| \beta_{k} \right|$ subject to $\sum_{k\geq1} \beta_{k}=0$. Here, $\lambda$ is the penalization parameter, and forces some of the $\boldsymbol{\beta}$-coefficients to go zero, particularly those that do not have a relationship with the genera/pathways and serves as a means to do variable selection. We have used coda_glmnet() function from R’s coda4microbiome package (6) . We have used the top 100 most abundant genera in the CODA-LASSO model.

We used the “BVSTEP” routine (7), an algorithm that searches for highest correlation between the distance matrices (Bray-Curtis distance) between two abundance tables, one original table with fixed number of OTUs, and one which is a copy of the same table, but is allowed to keep variable number of OTUs in the optimisation process. Through a permutation procedure, by systematically iterating through the subsets of these OTUs, the second table gets imploded down to the absolute minimal set OTUs that roughly conserve the same beta diversity distance between the samples as the full set of OTUs, thus we are only left with the main patterns that change between multiple categories (time points in our case). For this purpose, we have used bvStep() from R’s sinkr package (8). This analysis is complimentary to the differential analysis and identified the OTUs that were causing the major shifts in beta diversity.

To identify core microbiome, we have used the approach discussed in (9). The approach first ranks the OTUs by occupancy and their replicate consistency, and then calculates the minimal occupancy threshold dynamically by learning from the data. After ranking the OTUs, the subset of core taxa is constructed incrementally by adding one OTU at a time to the core set of OTUs, from highly prevalent to lowly prevalent ones. The contribution of the core subset to beta diversity is then calculated every time a new OTU becomes member of the core set using the Bray-Curtis distance in the equation, $C=1-\frac{BC_{core}}{BC_{all}}$. The authors have specified an approach to decide at what threshold of occupancy the core subset construction stops: where addition of an OTU does not cause more than 2% increase in the explanatory power by Bray-Curtis distance. Independently, a neutral model (9) is fitted to the “S” shaped abundance-occupancy distributions informing about the OTUs that are likely selected by the environment. These are obtained as those that fall outside the 95% confidence interval of the fitted model, and are inferred to be deterministically assembled, rather than neutrally selected, with those that are *above the model selected by the host environment* (represented by red colour), and those points below the model *are dispersal limited* (represented by blue colour).

To incorporate heterogeneity caused by time, we have used two approaches as per original author’s suggestion: a) a conservative and restrictive approach (no time-specific occupancy) where all discrete samples contribute equally to the calculation of occupancy, expressed as a proportion of 1 (or a percentage out of 100%), returning only those OTUs that are detected in every sample, and is sometimes biased towards more abundant OTUs; and a time specific approach, where occupancy is viewed as a detection within a particular time, such that as long as the OTUs is represented in each time (not necessarily in all replicates within that time), it is counted as occurring there. The latter approach is prone to returning false-positive core OTUs, however, on average, it picks up medium to low abundant, and low occupancy OTUs. We then used the neutral modelling approach to partition these core OTUs to those that are neutral, and those that are above/below the model fit (deterministically/assembled). To draw these OTUs, we have used the R’s metacoder package (10). In several figures, TSS+CLR represent Total Sum Scaling + Centralised Log Ratio transformed abundance counts.

To see if the microbes act as mediators between the treatment groups MS MAP- and MS MAP+, and the outcome variable *Disease Duration* (which we used after it being significant using the PERMANOVA analysis), we have employed a mediation analysis using R’s LDM package (11). The analysis also incorporates the confounders which are as follows: *Sex*, *Age*, *BMI*, *Time point*, *Weight change*, *Have children*, *Have pets*, *Smoker*, *Does work*, *Does sports*, *How much free time*, *How much sleep*, *Antibiotics, How often eating sleeps, How much drinking, Drink alcohol*, and *Probiotics*. The approach uses an inverse regression approach to regress the microbiome data at each taxon on to the treatment groups and the treatment group adjusted outcome whilst taking into account different confounders. Then, the *P*-values for testing the coefficients are used to test mediation at both the community (global) and individual taxon (OTUs) level. As per original authors’ recommendations, we have only used those OTUs that occupy at least 5 samples.

To analyses the categorical clinical parameters including the self-reported questionnaire, and to see if any two covariates have a relationship, we constructed a contingency table and used $\chi^{2}$ test of independence using chisq.test() function in R. Based on recommendations given in <http://www.sthda.com/english/wiki/chi-square-test-of-independence-in-r>, and where the $\chi^{2}$ test was significant, we then calculated the $\chi^{2}$ residuals and contribution for individual rows and columns of the contingency table. These were drawn using R’s corrplot (12) package where for a residual plot, positive values in cells specify an attraction (positive association; blue) between the corresponding row and column variables whilst negative values implies a repulsion (negative association; red) between the corresponding row and column variables.

**Supplementary Table S1**: **Summary statistics of meta data collected for samples (n=69): Set 1**. Categorical data (represented with ‘(n)’) with all possible outcomes along with the frequency of occurrence are also shown.

| **Groups** | **Total Samples**  **(n)** | **Timepoints**  **(n)** |
| --- | --- | --- |
|  |  |  |
| **HC MAP+** | 5 | T1:2 T2:3 |
| **HC MAP-** | 18 | T1:10 T2:8 |
| **MS MAP+** | 27 | T1:17  T2:10 |
| **MS MAP-** | 19 | T1:13  T2:5 |

**Supplementary Table S2**: **Summary statistics of meta data collected for participants (n=49): Set 2**. Description is similar to that of Supplementary Table S1. Here IQR stands for Inter Quartile Range.

| **Groups** | **Age (Years)** | | **Height (cm)** | | **Weight (Kg)** | | **BMI** | | **Disease duration**  **(Years)** | | **EDSS score** | | **Living area**  **(n)** | **Living environment**  **(n)** |
| --- | --- | --- | --- | --- | --- | --- | --- | --- | --- | --- | --- | --- | --- | --- |
|  | Median | IQR | Median | IQR | Median | IQR | Median | IQR | Median | IQR | Median | IQR |  |  |
| **HC MAP+** | 23 | 15.9-30.1 | 167 | 167-172 | 60 | 51-64 | 18.51 | 15.86-21.16 | NA | NA | NA | NA | Small city:3 | Coast:3 |
| **HC MAP-** | 36.7 | 15.1-58.3 | 166 | 163-178.2 | 67 | 63-79.75 | 25.28 | 21.07-29.49 | NA | NA | NA | NA | Big city:1  Medium city:2  Small city:8 | Coast:10  Countryside:1 |
| **MS MAP+** | 40.1 | 26.275-53.925 | 165 | 160-170 | 65 | 56.5-78.5 | 22.74 | 18.215-27.265 | 10.5 | 1.5-19.5 | 2 | 0.5-4.5 | Big city:2 Medium city:11 Small city:6 Village:1 | Coast:19 Countryside:1 |
| **MS MAP-** | 54.90 | 44.05-65.75 | 165 | 161-175.5 | 70 | 58.5-79. 5 | 22.83 | 16.82-28.84 | 13 | 7-19 | 3.5 | 0.375-6.625 | Big city:5 Medium city:6 Small city:3 Village:1 | Coast:15 |

**Supplementary Table S3**: **Summary statistics of meta data collected for participants (n=49): Set 3**. Description is similar to that of Supplementary Tables S1 & S2.

| **Groups** | **Sex**  **(n)** | **Children**  **(n)** | **Pets**  **(n)** | **Smoker**  **(n)** | **Travel Habit (n)** | **Work Routine (n)** | **Sports Activity (n)** |
| --- | --- | --- | --- | --- | --- | --- | --- |
| **HC MAP+** | Female:2  Male:1 | Yes:0  No:3 | Yes:0  No:3 | Yes:0  No:3 | 1 per year:1  No travel:2 | Sitting mostly:1  Sitting only:1  Standing mostly:1 | Yes:2  No:1 |
| **HC MAP-** | Female:6  Male:5 | Yes:4 No:7 | Yes:2 No:9 | Yes:1 No:10 | 1 per year:4  2-3 per year:1 No travel:6 | No work:1 Sitting mostly:3  Sitting only:5  Standing mostly:2 | Yes:2 No:9 |
| **MS MAP+** | Female:11  Male:9 | Yes:7 No:13 | Yes:7 No:13 | Yes:6 No:14 | 1 per year:1 No travel:19 | No work:9 Sitting mostly:1  Sitting only:4  Standing mostly:6 | Yes:6 No:14 |
| **MS MAP-** | Female:9  Male:6 | Yes:11 No:4 | Yes:7 No:13 | Yes:4 No:11 | 1 per year:1 No travel:14 | No work:6  Sitting mostly:1  Sitting only:2  Standing mostly:6 | Yes:8 No:7 |

**Supplementary Table S4**: **Summary statistics of meta data collected for participants (n=49): Set 4**. Description is similar to that of Supplementary Tables S1 & S2.

| **Groups** | **Leisure time (n)** | **Sleep Duration**  **(n)** | **Sleep Restfulness**  **(n)** | **Stressed** | | **Drugs Intake (n)** | **Meat Exclusion**  **(n)** |
| --- | --- | --- | --- | --- | --- | --- | --- |
|  |  |  |  | Median | IQR |  |  |
| **HC MAP+** | Normal:2  Sitting:1 | 6_7 hours:1  7_8 hours:2 | Yes:3  No:0 | 6 | 5-7 | Yes:0  No:3 | Yes:1  No:2 |
| **HC MAP-** | Active:2 Normal:4 Sitting:5 | 4_5 hours:1 5_6 hours:1 6_7 hours:7 7_8 hours:1 >8 hours:1 | Yes:7 No:4 | 8 | 6.5-9.5 | Yes:0  No:11 | Yes:4 No:7 |
| **MS MAP+** | Active:3 Inactive:3 Normal:8 Sitting:6 | 4-5 hours:4 5-6 hours:5 6-7 hours:8 7-8 hours:2 >8 hours:1 | Yes:8 No:12 | 7 | 5-9 | Yes:1 No:19 | Yes:13 No:7 |
| **MS MAP-** | Active:2 Inactive:2 Normal:6 Sitting:5 | 4-5 hours:4 5-6 hours:1 6-7 hours:5 7-8 hours:3 >8 hours:1 | Yes:3 No:12 | 8 | 2.5-13.5 | Yes:1 No:14 | Yes:7 No:8 |

**Supplementary Table S5**: **Summary statistics of meta data collected for participants (n=49): Set 5**. Description is similar to that of Supplementary Tables S1 & S2.

| **Groups** | **Fish consumption (n)** | **Vegetable consumption (n)** | **Sweet consumption**  **(n)** | **Drinking Water**  **(n)** | **Alcohol consumption (n)** | **Daily liquid consumption**  **(Water)**  **(n)** | **Daily liquid consumption**  **(Juice)**  **(n)** | **Daily liquid consumption**  **(soft drinks ,sugar)**  **(n)** |
| --- | --- | --- | --- | --- | --- | --- | --- | --- |
| **HC MAP+** | 1-2 per week:1  1 per week:2 | 1-2 per week:1  3-4 per week:1  Daily:1 | 1 per week:1  3-4 per week:1  Daily:1  <2 per month:1 | 1-1.5 liters:1  1.5-2 liters:2 | Never:3 | Yes:3  No:0 | Yes:0  No:3 | Yes:0  No:5 |
| **HC MAP-** | 1-2 per week:4 1 per 2 weeks:2 1 per week:3 3-4 per week:1 <2 per month:1 Never:1 | 1-2 per week:3  5-6 per week:1  Daily:7 | 1-2 per week:6  1 per 2 weeks:1  1 per week:1  3-4 per week:1  5-6 per week:1  Daily:1 <2 per month:1 | 1-1.5 liters:6 1.5-2 liters:2 >2 liters:1 <1 liter:2 | 1-2 per week:2 <5 months:2 Never:7 | Yes:10 No:1 | Yes:0  No:11 | Yes, without sugar:2 Yes, with sugar:1 No:15 |
| **MS MAP+** | 1-2 per week:9  1 per 2 weeks:1  1 per week:2  3-4 per week:1  <2 per month:7 | 1-2 per week:4 3-4 per week:3 5-6 per week:3 Daily:10 | 1-2 per week:5 1 per 2 weeks:1 1 per week:2 3-4 per week:4 5-6 per week:2 Daily:3 <2 per month:1 Never:2 | 1-1.5 liters:8 1.5-2liter:6 >2 liters:3 <1 liter:3 | 1-2 per week:9 3-4 per week:2 <5 months:3 Never:6 | Yes:20  No:0 | Yes:1 No:19 | Yes, without sugar:2 Yes, with sugar:2 No:23 |
| **MS MAP-** | 1-2 per week:5 1_per_2_weeks:3 1 per week:2 3-4 per week:1 Daily:1 <2 per month:3 | 1-2 per week:2 3-4 per week:3 Daily:10 | 1-2 per week:6 3-4 per week:4 5-6 per week:1 Daily:1 <2 per month:2 Never:1 | 1-1.5 liter:2 1.5-2 liter:5 >2 liters:3 <1 liter:5 | 1-2 per week:7 <5 months:3 Never:5 | Yes:14 No:1 | Yes:2 No:13 | Yes, without sugar:2 No:17 |

**Supplementary Table S6**: **Summary statistics of meta data collected for participants (n=49): Set 6**. Description is similar to that of Supplementary Tables S1 & S2.

| **Groups** | **Tea consumption**  **(Sugar)**  **(n)** | **Coffee consumption**  **(Sugar)**  **(n)** | **Probiotics consumption**  **(n)** | **Fermented food cheese**  **(n)** |
| --- | --- | --- | --- | --- |
| **HC MAP+** | No:3 | Yes, without sugar:2  No:1 | 6-12 months:1  Never:2 | Yes:2  No:1 |
| **HC MAP-** | Yes, without sugar:6  Yes, with sugar:1 No:4 | Yes, without sugar:5  Yes, with sugar:2  No:4 | >1 year:4 1-3 months:2  6-12 months:1  Never:4 | Yes:5  No:6 |
| **MS MAP+** | Yes, without sugar:3  No:17 | Yes, without sugar:11  No:9 | >1 year:4  3-6 months:2  Currently:1  Never:13 | Yes:8  No:12 |
| **MS MAP-** | Yes, without sugar:3 No:12 | Yes, without sugar:7  No:8 | >1 year:3  3-6 months:1  6-12 months:2  Currently:1  Never:8 | Yes:9  No:6 |

**Supplementary Table S7**: **Summary statistics of meta data collected for participants (n=49): Set 7**. Description is similar to that of Supplementary Tables S1 & S2.

| **Groups** | **Fermented food yogurt**  **(n)** | **Fermented food kefir**  **(n)** | **Fermented food miso**  **(n)** |
| --- | --- | --- | --- |
| **HC MAP+** | Yes:2  No:1 | No:3 | No:3 |
| **HC MAP-** | Yes:7 No:4 | No:11 | No:11 |
| **MS MAP+** | Yes:13 No:7 | Yes:1 No:19 | Yes:2 No:18 |
| **MS MAP-** | Yes:12 No:3 | Yes:0 No:11 | No:11 |

**Supplementary Table S8**: **Summary statistics of meta data collected for participants (n=49): Set 8**. Description is similar to that of Supplementary Tables S1 & S2.

| **Groups** | **Food supplements**  **(n)** | **Vitamins**  **(n)** | **Wellbeing**  **(n)** | | **Health**  **(n)** | | **Allergies**  **(n)** |
| --- | --- | --- | --- | --- | --- | --- | --- |
|  |  |  | Median | IQR | Median | IQR |  |
| **HC MAP+** | Yes:1  No:2 | Yes:1  No:2 | 8.0 | 6.5-9.5 | 8.0 | 5.5-10.5 | Yes:2  No:1 |
| **HC MAP-** | Yes:1 No:10 | Yes:1 No:10 | 7 | 5-7 | 7 | 6.5-7.5 | Yes:5 No:6 |
| **MS MAP+** | No:20 | No:20 | 7 | 6.75-7.25 | 8 | 7-9 | Yes:4 No:16 |
| **MS MAP-** | Yes:1 No:14 | Yes:1 No:14 | 7 | 6-8 | 7 | 6.5-7.5 | Yes:3 No:12 |

**Supplementary Table S9**: **Summary statistics of meta data collected for participants (n=49): Set 9**. Description is similar to that of Supplementary Tables S1 & S2.

| **Groups** | **Food intolerances lactose (n)** | **Food intolerances gluten (n)** | **house dust allergy**  **(n)** | **Nickel allergy**  **(n)** |
| --- | --- | --- | --- | --- |
| **HC MAP+** | Yes:1  No:2 | No:3 | No:3 | No:3 |
| **HC MAP-** | Yes:3 No:8 | Yes:1 No:10 | No:11 | Yes:1 No:10 |
| **MS MAP+** | Yes:2 No:18 | Yes:1 No:19 | Yes:2 No:18 | No:20 |
| **MS MAP-** | Yes:2 No:13 | Yes:1 No:14 | Yes:1 No:14 | Yes:1 No:14 |

**Supplementary Table S10**: **Summary statistics of meta data collected for participants (n=49): Set 10**. Description is similar to that of Supplementary Tables S1 & S2.

| **Groups** | **Antibiotics**  **(n)** | **Migraine**  **(n)** | **Migraine frequency**  **(n)** | **Cold**  **frequency**  **(n)** | **Indigestion constipation**  **(n)** |
| --- | --- | --- | --- | --- | --- |
| **HC MAP+** | 3-6 months:2  6-12 months:1 | No:5 | Never:3 | 2-3 per year:1  4-5 per year:1  <1 per year:2 | No:3 |
| **HC MAP-** | 1-3 months:1 >1 year:3 6-12 months:4  <1 month:2  Never:1 | No:14 No headache:3  Yes insecure:1 | 3-9 times:1 Never:10 | 2-3 per year:5 4-5 per year:1 >5 per year:1 <1 per year:4 | Yes >3 months:2 No:9 |
| **MS MAP+** | 1-3 months:4 >1 year:11 3-6 months:1 6-12 months:3 Never:1 | No:20 No headache:5  Yes insecure:2 | 3-9 times:1  Never:19 | 2-3 per year:2 4-5 per year:1 >5 per year:2 <1 per year:15 | No:17 Yes <3 months:1 Yes >3 months:2 |
| **MS MAP-** | >1 year:6 6-12 months:7 <1 month:2 | No:11  No headache:4  Yes diagnosed:2 Yes insecure:2 | 3-9 times:1  <3 times:2  Never:12 | 2-3 per year:3  >5 per year:1  <1 per year:11 | No:9  Yes >3 months:6 |

**Supplementary Table S11**: **Summary statistics of meta data collected for participants (n=49): Set 11**. Description is similar to that of Supplementary Tables S1 & S2.

| **Groups** | **Indigestion diarrhea**  **(n)** | **indigestion flatulence**  **(n)** | **Indigestion stomach pain**  **(n)** | **Bowel movement**  **(n)** |
| --- | --- | --- | --- | --- |
| **HC MAP+** | No:1  Yes <2 months:1  Yes >2 months:1 | No:3 | Yes:1  No:2 | 2-3 per day:1  Daily:1  Irregular:1 |
| **HC MAP-** | No:8 Yes <2 months:3 | Yes:1  No:10 | Yes:2  No:9 | 1 per 2 days:1  2-3 per day:1  Daily:4  Irregular:5 |
| **MS MAP+** | No:17  Yes >2 months:3 | Yes:2  No:18 | No:13  Yes:7 | 1 per 2 days:2  <3 per week:1  Daily:4  Irregular:13 |
| **MS MAP-** | No:15 | No:12 Yes:3 | No:11  Yes:4 | 1 per 2 days:1  <3 per week:1  >3 per day:1  Daily:3  Irregular:9 |

**Supplementary Table S12**: **Summary statistics of meta data collected for participants (n=49): Set 12**. Description is similar to that of Supplementary Tables S1 & S2.

| **Groups** | **Stool consistency** | | **Diseases other than MS**  **(n)** | **Medicine intake for other diseases**  **(n)** | **MS treatments**  **(TYSABRI)**  **(n)** | **MS treatments**  **(CLADRIBINA)**  **(n)** |
| --- | --- | --- | --- | --- | --- | --- |
|  | Median | IQR |  |  |  |  |
| **HC MAP+** | 4 | 2-6 | No:3 | No:3 | No:3 | No:3 |
| **HC MAP-** | 4 | 2-6 | Yes:1  No:10 | Yes:1  No:10 | No:11 | No:11 |
| **MS MAP+** | 3.5 | 2.5-4.5 | Yes:18  No:2 | Yes:17  No:3 | Yes:4  No:16 | Yes:1  No:19 |
| **MS MAP-** | 3 | 1-5 | Yes:13  No:2 | Yes:13  No:2 | Yes:3  No:12 | No:15 |

**Supplementary Table S13**: **Summary statistics of meta data collected for participants (n=49): Set 13**. Description is similar to that of Supplementary Tables S1 & S2.

| **Groups** | **MS treatments**  **(TECFIDERA)**  **(n)** | **MS treatments**  **(GILENYA)**  **(n)** | **MS treatments**  **(OCREVUS)**  **(n)** | **MS treatments**  **(IFNBETA)**  **(n)** | **MS treatments**  **(AUBAGIO)**  **(n)** | **MS treatments**  **(COPAXONE)**  **(n)** |
| --- | --- | --- | --- | --- | --- | --- |
| **HC MAP+** | No:3 | No:3 | No:3 | No:3 | No:3 | No:3 |
| **HC MAP-** | No:11 | No:11 | No:11 | No:11 | No:11 | No:11 |
| **MS MAP+** | Yes:5 No:15 | Yes:3 No:17 | Yes:1  No:19 | Yes:3 No:17 | No:20 | No:17 Yes:3 |
| **MS MAP-** | Yes:5 No:10 | No:15 | Yes:1 No:14 | Yes:2 No:13 | Yes:1 No:14 | Yes:2 No:13 |

**Supplementary Figure S1**. Relative proportions of at most top 25 abundant taxa at (A) genus, (B) family, and (C) phylum level, respectively. “Others” are collated taxa that did not make into the top 25 list.

**Supplementary Table S14**: PERMANOVA using different beta-diversity distances performed for clinical/demographic metadata using all the samples (HC MAP+, HC MAP-, MS MAP+, and MS MAP-). R^2^ value where significant (p<0.05) represents the percentage variability explained by the covariate.

| **Covariate** | **Bray-curtis** | **Unweighted UniFrac** | **Weighted UniFrac** | **Functional Hierarchical Meta-Storms** |
| --- | --- | --- | --- | --- |
| **Anthropometrie Parameters & Health Condition** | | | | |
| **Sex**  **(M/F)** | NS | NS | NS | R^2^=0.02418  *p-value* =0.048 * |
| **Age** | R^2^=0.01924  *p-value* = **0.018*** | NS | NS | NS |
| **Weight Change**  **(gain/loss /no change)** | NS | NS | NS | NS |
| **BMI** | NS | NS | NS | NS |
| **MAP Status**  **(MAP+/MAP-)** | R^2^=0.03874  *p-value*= **0.003 **** | R^2^=0.03741  *p-value*= **0.004 **** | R^2^= 0.05619  *p-value=* **0.023 *** | R^2=^ 0.05466  *p-value=* **0.007 **** |
| **Appendectomy**  **(Y/N)** | NS | NS | NS | NS |
| **Medicine Intake**  **(Y/N)** | R^2^= 0.01857  *p-value*=**0.036 *** | R^2=^ 0.01907  *p-value=* **0.023 *** | NS | R^2^= 0.04205  *p-value=* 0.006 ** |
| **Medicinal Group** | | | | |
| AUBAGIO  **(Y/N)** | R^2^= 0.0228  *p-value=* **0.013 *** | R^2=^ 0.02284  *p-value=* **0.031 *** | R^2^= 0.03243  *p-value=* **0.036 *** | NS |
| CLADRIBINA  **(Y/N)** | R^2^= 0.02712  *p-value=* **0.003 **** | NS | NS | NS |
| COPAXONE  **(Y/N)** | R^2^= 0.02155  *p-value=* **0.004 **** | NS | R^2^= 0.03148  *p-value=* **0.025 *** | NS |
| GILENYA  **(Y/N)** | R^2^= 0.0204  *p-value=* **0.014 *** | NS | NS | NS |
| IFN-BETA  **(Y/N)** | NS | NS | NS | NS |
| OCREVUS  **(Y/N)** | NS | NS | NS | NS |
| TECFIDERA  **(Y/N)** | R^2=^ 0.02131  *p-value=* **0.005 **** | NS | NS | R^2=^ 0.02603  *p-value=***0.028 *** |
| TYSABRI  **(Y/N)** | NS | NS | NS | NS |
| **Antibiotics effect**   1. 1_3_months 2. 3_6_months 3. 6_12_months 4. 1_more_year | R^2=^ 0.09358  *p-value=* **0.001 ***** | R^2=^ 0.0866  *p-value=* **0.001 ***** | R^2=^ 0.13277  *p-value=* **0.002 **** | R^2=^ 0.11576  *p-value=* **0.008 **** |
| **Stool consistency**  **(Bristol scale)** | R^2^= 0.023  *p-value=* **0.002 **** | R^2=^ 0.02766  *p-value=* **0.023 *** | NS | R^2^= 0.02766  *p-value=* 0.032 * |
| **Bowel movement**  **(Y/N)** | R^2=^ 0.07965  *p-value=* **0.05 *** | R^2=^ 0.08177  *p-value=* **0.031 *** | R^2=^ 0.10375  *p-value=* **0.036 *** | NS |
| **Indigestion constipation**  **(Y/N)** | R^2^= 0.03428  *p-value=* 0.042 * | NS | NS | NS |
| **Indigestion diarrhea**  **(Y/N)** | NS | R^2=^0.04103  *p-value=* **0.002 **** | NS | NS |
| **Indigestion flatulence**  **(Y/N)** | NS | NS | NS | NS |
| **Indigestion stomach pain**  **(Y/N)** | NS | NS | NS | NS |
| **Timepoint**  **(T1/T2)** | NS | NS | NS | NS |
| **Cold occurrence**  **(Y/N)** | R^2=^ 0.05195  *p-value=* **0.013 *** | R^2=^ 0.05944  *p-value=* **0.002 **** | R^2=^ 0.0776  *p-value=* **0.016 *** | R^2=^ 0.06337  *p-value=* **0.036 *** |
| **Disease status**  **(MS/Healthy)** | R^2=^ 0.02049  *p-value=* **0.01 **** | R^2^= 0.01969  *p-value=* **0.011 *** | NS | NS |
| **Migraine**  **(Y/N)** | NS | NS | NS | NS |
| **Migraine severity**   1. less_3_times 2. 3 and 9 times 3. More than10 times | NS | NS | NS | NS |
| **Allergies**  **(Y/N)** | NS | NS | NS | NS |
| **Allergies**  **(House dust)**  **(Y/N)** | NS | NS | NS | NS |
| **Allergies**  **(Nickel)**  **(Y/N)** | NS | NS | NS | NS |
| **Food intolerance**  **(Gluten)**  **(Y/N)** | NS | NS | NS | NS |
| **Food intolerance**  **(lactose)**  **(Y/N)** | NS | NS | NS | NS |
| **Environment (Life Condition)** | | | | |
| **Travelling**   1. no_travel 2. 1_per_year 3. 2_3_per_year 4. More than 3 times a year | NS | R^2=^ 0.03459  *p-value=* **0.022 *** | R^2=^ 0.0515  *p-value=* **0.041 *** |  |
| **Have children**  **(Y/N)** | R^2=^ 0.01768  *p-value=* **0.049 *** | R^2=^ 0.01879  *p-value=* **0.014 *** | R^2=^ 0.02973  *p-value=* **0.034 *** | R^2=^ 0.0315  *p-value=* **0.01 **** |
| **Work**  **(Work type)** | R^2=^ 0.07077  *p-value=* **0.008 **** | R^2=^ 0.06674  *p-value=* **0.024 *** | R^2=^ 0.09751  *p-value=* **0.014 *** |  |
| **Living environment**  **(Countryside/coast)** | NS | NS | NS | NS |
| **Living location**  **(village/small city/large city)** | NS | NS | NS | NS |
| **Pets**  **(Y/N)** | R^2=^ 0.01871  *p-value=* **0.021 *** | NS | R^2=^ 0.03252  *p-value=* **0.026 *** | NS |
| **Lifestyle (Nutrition, Sleep, Habits)** | | | | |
| **Sleep duration**   1. 4_5_hours 2. 6_7_hours 3. 7_8_hours 4. 8_more_hours | R^2=^ 0.08055  *p-value=* **0.02 *** | R^2=^ 0.08147  *p-value=* **0.015 *** | NS | NS |
| **Restful sleep**  **(Y/N)** | NS | NS | NS | NS |
| **Leisure time**  **(normal/sitting/active)** | NS | NS | NS | NS |
| **Sports**  **(Y/N)** | NS | NS | NS | NS |
| **Smoker**  **(Y/N)** | R^2=^ 0.0206  *p-value=* **0.011 *** | NS | NS | R^2=^ 0.03544  *p-value=* **0.006 **** |
| **Drugs intake**  **(Y/N)** | NS | NS | NS | R^2=^ 0.04205  *p-value=* **0.006 **** |
| **Alcohol consumption**  **(Y/N)** | NS | NS | NS | NS |
| **Probiotics**  **(Y/N)** | R^2=^ 0.08397  *p-value=* **0.005 **** | NS | NS | NS |
| **Probiotic fermented food**  **(cheese)**  **(Y/N)** | NS | NS | NS | NS |
| **Probiotic fermented food**  **(Kefir)**  **(Y/N)** | R^2=^ 0.02449  *p-value=* **0.003 **** | R^2=^ 0.01996  *p-value=* **0.047 *** | NS | NS |
| **Probiotic fermented food**  **(miso)**  **(Y/N)** | NS | NS | NS | NS |
| **Probiotic fermented food**  **(yogurt)**  **(Y/N)** | NS | R^2=^ 0.01763  *p-value=* **0.048 *** | NS | NS |
| **Vegetables intake**   1. daily 2. 1_2_per_week 3. 3_4_per_week 4. 5_6_per_week 5. less_2_month 6. never | NS | NS | NS | NS |
| **Coffee Intake**  **(With sugar)**  **(Y/N)** | R^2=^ 0.0364  *p-value=*  **0.016 *** | R^2=^ 0.0385  *p-value=* **0.003 **** | R^2=^ 0.05989  *p-value=* **0.011 *** | NS |
| **Water drinking capacity**   1. less_1_literb) 2. 1_1.5_liter 3. 1.5_2_liter 4. 2_more_liter | NS | NS | NS | NS |
| **Drinking**  **(juice)**  **(Y/N)** | NS | NS | NS | NS |
| **Drinking**  **(water)** | NS | NS | NS | NS |
| **Soft drinks intake**  **(Y/N)** | NS | NS | NS | NS |
| **Tea intake**  **(With sugar)**  **(Y/N)** | R^2=^ 0.04476  *p-value=* **0.003 **** | R^2=^ 0.04181  *p-value=* **0.011 *** | R^2=^ 0.07182  *p-value=* **0.009 **** | R^2=^ 0.06904  *p-value=* **0.008 **** |
| **Sweets intake**   1. 1_2_per_week 2. 3_4_per_week 3. 5_6_per_week 4. Daily 5. less_2_month 6. 1_per_week | NS | NS | NS | NS |
| **Fish intake**   1. daily 2. 1_2_per_week 3. 3_4_per_week 4. 5_6_per_week 5. less_2_month 6. never | R^2=^ 0.09853  *p-value=* **0.014 *** | R^2=^ 0.09551  *p-value=* **0.035 *** | R^2=^ 0.13564  *p-value=* **0.01 **** | NS |
| **Meat intake**   1. daily 2. 1_2_per_week 3. 3_4_per_week 4. 5_6_per_week 5. less_2_month 6. never | R^2=^ 0.11365  *p-value=* **0.024 *** | NS | R^2=^ 0.13636  *p-value=* **0.041 *** | NS |

**Supplementary Table S15**: Similar to Supplementary Table S14, but PERMANOVA is now performed on a subset of the samples (MS MAP+, and MS MAP- cohort only) where additional clinical data was available. R^2^ value where significant (p<0.05) represents the percentage variability explained by the covariate.

| **Covariate** | **Bray-curtis** | **Unweighted UniFrac** | **Weighted UniFrac** | **Functional Hierarchical Meta-Storms** |
| --- | --- | --- | --- | --- |
| **Anthropometrie Parameters & Health Condition** | | | | |
| **Sex**  **(M/F)** | R^2=^ 0.02751  *p-value*= **0.05 *** | R^2=^ 0.04229  *p-value*= **0.027 *** | NS | R^2=^ 0.04271  *p-value*= **0.029 *** |
| **Age** | NS | NS | NS | NS |
| **Weight Change**  **(gain/loss /no change)** | NS | NS | NS | NS |
| **BMI** | NS | NS | NS | NS |
| **MAP Status**  **(MAP+/MAP-)** | R^2=^ 0.02794  *p-value*= **0.039 *** | R^2=^ 0.04504  *p-value*= **0.018 *** | R^2=^ 0.02886  *p-value=* **0.017 *** | R^2=^ 0.04991  *p-value=* **0.012 *** |
| **EDSS Score** | NS | NS | NS | NS |
| **Disease Duration** | R^2=^ 0.03192  *p-value=* **0.013 *** | R^2=^ 0.0284  *p-value=* **0.049 *** | NS | NS |
| **Appendectomy**  **(Y/N)** | NS | NS | NS | NS |
| **Medicine Intake**  **(Y/N)** | NS | NS | NS | NS |
| **Medicinal Group** | | | | |
| AUBAGIO  **(Y/N)** | R^2=^ 0.03471  *p-value=* **0.012 *** | NS | R^2=^ 0.0365  *p-value=* **0.047 *** | R^2=^ 0.04914  *p-value=* **0.038 *** |
| CLADRIBINA  **(Y/N)** | R^2=^ 0.03968  *p-value=***0.001 ***** | NS | NS | R^2=^ 0.04624  *p-value=* **0.046 *** |
| COPAXONE  **(Y/N)** | R^2=^ 0.03292  *p-value=***0.01 **** | NS | R^2=^ 0.02801  *p-value=* **0.039 *** | R^2=^ 0.05001  *p-value=* **0.013 *** |
| GILENYA  **(Y/N)** | R^2=^ 0.03022  *p-value=* **0.028 *** | NS | NS | NS |
| IFN-BETA  **(Y/N)** | NS | NS | NS | NS |
| OCREVUS  **(Y/N)** | NS | NS | NS | NS |
| TECFIDERA  **(Y/N)** | R^2=^ 0.0337  *p-value=* **0.005 **** | NS | NS | NS |
| TYSABRI  **(Y/N)** | NS | NS | NS | NS |
| **Antibiotics effect**   1. 1_3_months 2. 3_6_months 3. 6_12_months 4. 1_more_year | R^2=^ 0.12152  *p-value=* **0.047 *** | NS | NS | NS |
| **Stool consistency**  **(Bristol scale)** | R^2=^ 0.0303  *p-value=* **0.011 *** | NS | NS | NS |
| **Bowel movement**  **(Y/N)** | NS | NS | NS | NS |
| **Indigestion constipation**  **(Y/N)** | NS | NS | NS | NS |
| **Indigestion diarrhea**  **(Y/N)** | NS | NS | NS | NS |
| **Indigestion flatulence**  **(Y/N)** | NS | NS | NS | NS |
| **Indigestion stomach pain**  **(Y/N)** | NS | NS | NS | NS |
| **Timepoint**  **(T1/T2)** | NS | NS | NS | NS |
| **Cold occurrence**  **(Y/N)** | NS | NS | NS | NS |
| **Disease status**  **(MS/Healthy)** | NS | NS | NS | NS |
| **Migraine**  **(Y/N)** | NS | NS | NS | NS |
| **Migraine severity**   1. less_3_times 2. 3 and 9 times 3. More than10 times | NS | NS | NS | NS |
| **Allergies**  **(Y/N)** | NS | NS | NS | NS |
| **Allergies**  **(House dust)**  **(Y/N)** | NS | NS | NS | NS |
| **Allergies**  **(Nickel)**  **(Y/N)** | NS | NS | NS | NS |
| **Food intolerance**  **(Gluten)**  **(Y/N)** | NS | NS | NS | NS |
| **Food intolerance**  **(lactose)**  **(Y/N)** | NS | NS | NS | NS |
| **Environment (Life Condition)** | | | | |
| **Travelling**   1. no_travel 2. 1_per_year 3. 2_3_per_year 4. More than 3 times a year | NS | R^2=^ 0.03459  *p-value=* **0.02 *** | NS |  |
| **Have children**  **(Y/N)** | R^2=^ 0.03238  *p-value=* **0.008 **** | R^2=^ 0.06058  *p-value=* **0.004 **** | R^2^ =0.03602  *p-value=* **0.001 ***** | R^2=^ 0.07523  *p-value=* **0.001 ***** |
| **Work**  **(Work type)** | R^2=^  0.10138  *p-value=* **0.028 *** | NS | NS |  |
| **Living environment**  **(Countryside/coast)** | NS | NS | NS | NS |
| **Living location**  **(village/small city/large city)** | NS | NS | NS | NS |
| **Pets**  **(Y/N)** | NS | NS | NS | NS |
| **Lifestyle (Nutrition, Sleep, Habits)** | | | | |
| **Sleep duration**   1. 4_5_hours 2. 6_7_hours 3. 7_8_hours 4. 8_more_hours | R^2=^ 0.12309  *p-value=* **0.025 *** | NS | R^2=^ 0.12537  *p-value=* **0.029 *** | NS |
| **Restful sleep**  **(Y/N)** | NS | NS | NS | NS |
| **Leisure time**  **(normal/sitting/active)** | R^2=^0.07408  *p-value=* **0.043 *** | NS | R^2=^0.07374  *p-value=* **0.03 *** | NS |
| **Sports**  **(Y/N)** | NS | NS | R^2=^0.03359  *p-value=* **0.005 **** | NS |
| **Smoker**  **(Y/N)** | R^2=^ 0.02891  *p-value=* **0.029 *** | R^2=^ 0.04558  *p-value=* **0.009 **** | NS | NS |
| **Drugs intake**  **(Y/N)** | NS | R^2=^0.06106  *p-value=* ***0.009 ***** | NS | NS |
| **Alcohol consumption**  **(Y/N)** | NS | NS | NS | NS |
| **Probiotics**  **(Y/N)** | R^2^= 0.09997  *p-value=* **0.031 *** | NS | NS | NS |
| **Probiotic fermented food**  **(cheese)**  **(Y/N)** | NS | NS | R^2=^ 0.02972  *p-value=* **0.012 *** | NS |
| **Probiotic fermented food**  **(Kefir)**  **(Y/N)** | R^2^= 0.03615  *p-value=* **0.003 **** | NS | NS | NS |
| **Probiotic fermented food**  **(miso)**  **(Y/N)** | NS | NS | NS | NS |
| **Probiotic fermented food**  **(yogurt)**  **(Y/N)** | NS | NS | NS | NS |
| **Vegetables intake**   1. Daily 2. 1_2_per_week 3. 3_4_per_week 4. 5_6_per_week 5. less_2_month 6. never | NS | NS | NS | NS |
| **Coffee Intake**  **(With sugar)**  **(Y/N)** | NS | NS | NS | NS |
| **Water drinking capacity**   1. less_1_literb) 2. 1_1.5_liter 3. 1.5_2_liter 4. 2_more_liter | NS | NS | NS | NS |
| **Drinking**  **(juice)**  **(Y/N)** | NS | NS | NS | NS |
| **Drinking**  **(water)** | NS | NS | NS | NS |
| **Soft drinks intake**  **(Y/N)** | NS | NS | NS | NS |
| **Tea intake**  **(With sugar)**  **(Y/N)** | NS | NS | NS | NS |
| **Sweets intake**   1. 1_2_per_week 2. 3_4_per_week 3. 5_6_per_week 4. Daily 5. less_2_month 6. 1_per_week | NS | NS | NS | NS |
| **Fish intake**   1. Daily 2. 1_2_per_week 3. 3_4_per_week 4. 5_6_per_week 5. less_2_month   never | NS | NS | NS | NS |
| **Meat intake**   1. daily 2. 1_2_per_week 3. 3_4_per_week 4. 5_6_per_week 5. less_2_months 6. never | NS | NS | NS | R^2=^ 0.21076  *p-value=* **0.028 *** |

**Supplementary Figure S2**: Taxonomic coverage of core microbiome for HC cohort using different occupancy models (HC MAP- T1, HC MAP- T2, HC MAP+ T1, and HC MAP+ T2) displayed in Figure 3. The key shown in lower right side is as follows: colour (annotation on the left side of the key) represents the abundance of OTUs; whilst the width of the key represents the number of unique OTUs (annotation on the right of the key) and is size of the nodes. HC All is the collated taxonomic coverage tree of all the occupancy models added together.

**Supplementary Figure S3**: Taxonomic coverage of core microbiome for MS cohort using different occupancy models (MS MAP- T1, MS MAP- T2, MS MAP+ T1, and MS MAP+ T2) displayed in Figure 3. The key shown in lower right side is as follows: colour (annotation on the left side of the key) represents the abundance of OTUs; whilst the width of the key represents the number of unique OTUs (annotation on the right of the key) and is size of the nodes. MS All is the collated taxonomic coverage tree of all the occupancy models added together.

**Supplementary Figure S4**: Expression of significant OTUs identified from the BVSTEP routine with samples classified as HC, MAP-, and MAP+. The information, and the taxonomy of OTUs from the top subsets is given below the figure with the correlation between Bray–Curtis distances between a particular subset and the full OTU table given in parenthesis. For normalisation of OTU counts, TSS +CLR normalisation is used.

**Supplementary Table S16**: For testing mediation effects of the microbiome that mediate the effect of the exposure (MAP Status) on the outcome (Disease Duration), we have used the formula ‘abund_table | (**set of confounders**) ~ (**exposure + outcome**)’ in the function ldm() by setting test.mediation=TRUE and test.omni3=TRUE. Our exact formula is ‘abund_table | (**sex+Age+BMI+Time_Point+Weight_Change+Children+Pets+Smoker+Work+Sports+free_time+sleep_how_much+antibiotics+sweets_how_often+drinking_how_much+alcohol+probiotics**) ~ **MAP_Status**+**Disease_duration**’. Based on global tests, microbiome plays a mediating role using majority of the tests: Global test based on the frequency-scale data (**freq**; p = 0.0239); Global test based on arcsin-root-transformed frequency data (**tran**; p = *N.S.*); Global test based on presence-absence data (**pa**; p = 0.0042); Global test based on the Harmonic-mean p-value combination method (**harmonic**; p = 0.0041); Global test based on Fisher p-value combination (fisher; p = *N.S.*); Global test based on the omnibus statistics in LDM-omni (**omni**; p = 0.0256); Global test based on omnibus test LDM-omni3 (**omni3**; p=0.0106). At local level, whichever OTUs are playing a mediating role, selected by the procedure, their Q values (after adjusting for multiple comparisons for a given test) are shown, along with their taxonomy. Note that those where Q-values are significant (p<0.05) for both Covariate 1 and Covariate 2 have a relationship from exposure to outcome, and are represented in grey background. The abundance profile of these mediating OTUs for MAP status (MAP-, MAP+) is given in Supplementary Figure S10.

| Mediating OTUs | Covariate 1: MAP Status (Q-value) | Covariate 2: Disease Duration (Q-value) | OTU-specific test | Kingdom | Phylum | Class | Order | Family | Genus | Species |
| --- | --- | --- | --- | --- | --- | --- | --- | --- | --- | --- |
| OTU_1 | 0.00028259 | 0.19954014 | pa | Bacteria | Firmicutes | Clostridia | Oscillospirales | Ruminococcaceae | Faecalibacterium | |
| OTU_1056 | 0.00056416 | 0.209375 | pa | Bacteria | Firmicutes | Clostridia | Oscillospirales | Ruminococcaceae | |  |
| OTU_109 | 0.01764214 | 0.22572022 | pa | Bacteria | Bacteroidota | Bacteroidia | Bacteroidales | Marinifilaceae | Odoribacter |  |
| OTU_10932 | 0.00028259 | 0.00209368 | pa | Bacteria | Proteobacteria | Gammaproteobacteria | Burkholderiales | Sutterellaceae | Sutterella | Sutterella_wadsworthensis |
| OTU_10932 | 0.00025147 | 0.0015952 | omni3 | Bacteria | Proteobacteria | Gammaproteobacteria | Burkholderiales | Sutterellaceae | Sutterella | Sutterella_wadsworthensis |
| OTU_1106 | 0.04282155 | 0.01641498 | freq | Bacteria | Firmicutes | Clostridia | Lachnospirales | Lachnospiraceae | |  |
| OTU_1106 | 0.06313437 | 0.04889564 | omni | Bacteria | Firmicutes | Clostridia | Lachnospirales | Lachnospiraceae | |  |
| OTU_1106 | 0.00025147 | 0.05261093 | omni3 | Bacteria | Firmicutes | Clostridia | Lachnospirales | Lachnospiraceae | |  |
| OTU_116 | 0.0445955 | 0.09134082 | omni3 | Bacteria | Bacteroidota | Bacteroidia | Bacteroidales | Prevotellaceae | Paraprevotella | |
| OTU_1179 | 0.00028259 | 0.00209368 | pa | Bacteria | Verrucomicrobiota | Verrucomicrobiae | Verrucomicrobiales | Akkermansiaceae | Akkermansia | uncultured_bacterium |
| OTU_1179 | 0.00025147 | 0.0015952 | omni3 | Bacteria | Verrucomicrobiota | Verrucomicrobiae | Verrucomicrobiales | Akkermansiaceae | Akkermansia | uncultured_bacterium |
| OTU_12024 | 0.00028259 | 0.07470185 | pa | Bacteria | Firmicutes | Clostridia | Oscillospirales | Ruminococcaceae | Subdoligranulum | uncultured_bacterium |
| OTU_12024 | 0.00025147 | 0.19016284 | omni3 | Bacteria | Firmicutes | Clostridia | Oscillospirales | Ruminococcaceae | Subdoligranulum | uncultured_bacterium |
| OTU_12137 | 0.00028259 | 0.00209368 | pa | Bacteria | Bacteroidota | Bacteroidia | Bacteroidales | Bacteroidaceae | Bacteroides |  |
| OTU_12137 | 0.00025147 | 0.0015952 | omni3 | Bacteria | Bacteroidota | Bacteroidia | Bacteroidales | Bacteroidaceae | Bacteroides |  |
| OTU_1222 | 0.0748494 | 0.14795833 | omni3 | Bacteria | Bacteroidota | Bacteroidia | Bacteroidales | Barnesiellaceae | Barnesiella | uncultured_bacterium |
| OTU_1231 | 0.48762206 | 0.63134615 | freq | Bacteria | Firmicutes | Clostridia | Lachnospirales | Lachnospiraceae | Blautia |  |
| OTU_1231 | 0.01385883 | 0.10382669 | omni3 | Bacteria | Firmicutes | Clostridia | Lachnospirales | Lachnospiraceae | Blautia |  |
| OTU_125 | 0.00028259 | 0.0438027 | pa | Bacteria | Firmicutes | Clostridia | Lachnospirales | Lachnospiraceae | [Eubacterium]_xylanophilum_group | |
| OTU_125 | 0.00025147 | 0.08946574 | omni3 | Bacteria | Firmicutes | Clostridia | Lachnospirales | Lachnospiraceae | [Eubacterium]_xylanophilum_group | |
| OTU_1261 | 0.00028259 | 0.06010355 | pa | Bacteria | Firmicutes | Clostridia | Oscillospirales | Ruminococcaceae | uncultured | uncultured_bacterium |
| OTU_1261 | 0.00025147 | 0.12402444 | omni3 | Bacteria | Firmicutes | Clostridia | Oscillospirales | Ruminococcaceae | uncultured | uncultured_bacterium |
| OTU_129 | 0.00028259 | 0.04522809 | pa | Bacteria | Cyanobacteria | Vampirivibrionia | Gastranaerophilales | Gastranaerophilales | Gastranaerophilales | |
| OTU_129 | 0.00025147 | 0.08946574 | omni3 | Bacteria | Cyanobacteria | Vampirivibrionia | Gastranaerophilales | Gastranaerophilales | Gastranaerophilales | |
| OTU_1294 | 0.00028259 | 0.00209368 | pa | Bacteria | Firmicutes | Clostridia | Lachnospirales | Lachnospiraceae | [Eubacterium]_xylanophilum_group | |
| OTU_1294 | 0.00025147 | 0.0015952 | omni3 | Bacteria | Firmicutes | Clostridia | Lachnospirales | Lachnospiraceae | [Eubacterium]_xylanophilum_group | |
| OTU_13107 | 0.00251618 | 0.2305786 | pa | Bacteria | Bacteroidota | Bacteroidia | Bacteroidales | Bacteroidaceae | Bacteroides |  |
| OTU_13373 | 0.00028259 | 0.0438027 | pa | Bacteria | Firmicutes | Clostridia | Lachnospirales | Lachnospiraceae | |  |
| OTU_13373 | 0.00025147 | 0.09382654 | omni3 | Bacteria | Firmicutes | Clostridia | Lachnospirales | Lachnospiraceae | |  |
| OTU_1364 | 0.09793445 | 0.15999025 | omni3 | Bacteria | Bacteroidota | Bacteroidia | Bacteroidales | Prevotellaceae | Paraprevotella | |
| OTU_14079 | 0.00028259 | 0.04522809 | pa | Bacteria | Proteobacteria | Alphaproteobacteria | Rhodospirillales | uncultured | uncultured | gut_metagenome |
| OTU_14079 | 0.00025147 | 0.0975899 | omni3 | Bacteria | Proteobacteria | Alphaproteobacteria | Rhodospirillales | uncultured | uncultured | gut_metagenome |
| OTU_141 | 0.00028259 | 0.0438027 | pa | Bacteria | Firmicutes | Negativicutes | Acidaminococcales | Acidaminococcaceae | Acidaminococcus | |
| OTU_141 | 0.00025147 | 0.10624595 | omni3 | Bacteria | Firmicutes | Negativicutes | Acidaminococcales | Acidaminococcaceae | Acidaminococcus | |
| OTU_1425 | 0.00028259 | 0.0438027 | pa | Bacteria | Firmicutes | Clostridia | Lachnospirales | Lachnospiraceae | [Eubacterium]_hallii_group | uncultured_bacterium |
| OTU_1425 | 0.00025147 | 0.09060227 | omni3 | Bacteria | Firmicutes | Clostridia | Lachnospirales | Lachnospiraceae | [Eubacterium]_hallii_group | uncultured_bacterium |
| OTU_1432 | 0.00028259 | 0.04302184 | pa | Bacteria | Bacteroidota | Bacteroidia | Bacteroidales | Bacteroidaceae | Bacteroides |  |
| OTU_1432 | 0.00025147 | 0.08946574 | omni3 | Bacteria | Bacteroidota | Bacteroidia | Bacteroidales | Bacteroidaceae | Bacteroides |  |
| OTU_14507 | 0.00028259 | 0.00209368 | pa | Bacteria | Firmicutes | Clostridia | Lachnospirales | Lachnospiraceae | Anaerostipes | |
| OTU_14507 | 0.00025147 | 0.0015952 | omni3 | Bacteria | Firmicutes | Clostridia | Lachnospirales | Lachnospiraceae | Anaerostipes | |
| OTU_14580 | 0.00028259 | 0.00209368 | pa | Bacteria | Bacteroidota | Bacteroidia | Bacteroidales | Bacteroidaceae | Bacteroides |  |
| OTU_14580 | 0.00025147 | 0.0015952 | omni3 | Bacteria | Bacteroidota | Bacteroidia | Bacteroidales | Bacteroidaceae | Bacteroides |  |
| OTU_14671 | 0.00028259 | 0.00209368 | pa | Bacteria | Bacteroidota | Bacteroidia | Bacteroidales | Bacteroidaceae | Bacteroides |  |
| OTU_14671 | 0.00025147 | 0.0015952 | omni3 | Bacteria | Bacteroidota | Bacteroidia | Bacteroidales | Bacteroidaceae | Bacteroides |  |
| OTU_148 | 0.00028259 | 0.04274606 | pa | Bacteria | Bacteroidota | Bacteroidia | Bacteroidales | Rikenellaceae | Alistipes |  |
| OTU_148 | 0.00025147 | 0.08946574 | omni3 | Bacteria | Bacteroidota | Bacteroidia | Bacteroidales | Rikenellaceae | Alistipes |  |
| OTU_15009 | 0.00028259 | 0.00209368 | pa | Bacteria | Actinobacteriota | Actinobacteria | Actinomycetales | Actinomycetaceae | Actinomyces | Schaalia_odontolytica |
| OTU_15009 | 0.00025147 | 0.0015952 | omni3 | Bacteria | Actinobacteriota | Actinobacteria | Actinomycetales | Actinomycetaceae | Actinomyces | Schaalia_odontolytica |
| OTU_15059 | 0.00028259 | 0.04274606 | pa | Bacteria | Firmicutes | Clostridia | Oscillospirales | Ruminococcaceae | Faecalibacterium | |
| OTU_15059 | 0.00025147 | 0.08946574 | omni3 | Bacteria | Firmicutes | Clostridia | Oscillospirales | Ruminococcaceae | Faecalibacterium | |
| OTU_15112 | 0.00028259 | 0.00209368 | pa | Bacteria | Firmicutes | Clostridia | Lachnospirales | Lachnospiraceae | |  |
| OTU_15112 | 0.00025147 | 0.0015952 | omni3 | Bacteria | Firmicutes | Clostridia | Lachnospirales | Lachnospiraceae | |  |
| OTU_15779 | 0.00028259 | 0.0438027 | pa | Bacteria | Firmicutes | Clostridia | Lachnospirales | Lachnospiraceae | Fusicatenibacter | |
| OTU_15779 | 0.00025147 | 0.08946574 | omni3 | Bacteria | Firmicutes | Clostridia | Lachnospirales | Lachnospiraceae | Fusicatenibacter | |
| OTU_15798 | 0.00028259 | 0.00209368 | pa | Bacteria | Cyanobacteria | Vampirivibrionia | Gastranaerophilales | Gastranaerophilales | Gastranaerophilales | Acinetobacter_sp. |
| OTU_15798 | 0.00025147 | 0.0015952 | omni3 | Bacteria | Cyanobacteria | Vampirivibrionia | Gastranaerophilales | Gastranaerophilales | Gastranaerophilales | Acinetobacter_sp. |
| OTU_1588 | 0.00028259 | 0.00209368 | pa | Bacteria | Firmicutes | Clostridia | Lachnospirales | Lachnospiraceae | |  |
| OTU_1588 | 0.00025147 | 0.0015952 | omni3 | Bacteria | Firmicutes | Clostridia | Lachnospirales | Lachnospiraceae | |  |
| OTU_15911 | 0.00028259 | 0.00209368 | pa | Bacteria | Firmicutes | Clostridia | Lachnospirales | Lachnospiraceae | |  |
| OTU_15911 | 0.00025147 | 0.0015952 | omni3 | Bacteria | Firmicutes | Clostridia | Lachnospirales | Lachnospiraceae | |  |
| OTU_15983 | 0.00028259 | 0.07645899 | pa | Bacteria | Firmicutes | Clostridia | Lachnospirales | Lachnospiraceae | |  |
| OTU_15983 | 0.00025147 | 0.17661187 | omni3 | Bacteria | Firmicutes | Clostridia | Lachnospirales | Lachnospiraceae | |  |
| OTU_1629 | 0.00028259 | 0.00209368 | pa | Bacteria | Firmicutes | Clostridia | Lachnospirales | Lachnospiraceae | |  |
| OTU_1629 | 0.00025147 | 0.0015952 | omni3 | Bacteria | Firmicutes | Clostridia | Lachnospirales | Lachnospiraceae | |  |
| OTU_166 | 0.00028259 | 0.0438027 | pa | Bacteria | Proteobacteria | Gammaproteobacteria | Burkholderiales | Sutterellaceae | Sutterella | gut_metagenome |
| OTU_166 | 0.00025147 | 0.09594416 | omni3 | Bacteria | Proteobacteria | Gammaproteobacteria | Burkholderiales | Sutterellaceae | Sutterella | gut_metagenome |
| OTU_16658 | 0.00028259 | 0.00209368 | pa | Bacteria | Firmicutes | Clostridia | Lachnospirales | Lachnospiraceae | Anaerostipes | uncultured_bacterium |
| OTU_16658 | 0.00025147 | 0.0015952 | omni3 | Bacteria | Firmicutes | Clostridia | Lachnospirales | Lachnospiraceae | Anaerostipes | uncultured_bacterium |
| OTU_16721 | 0.00028259 | 0.00209368 | pa | Bacteria | Proteobacteria | Alphaproteobacteria | Rhodospirillales | uncultured | uncultured | Azospirillum_sp. |
| OTU_16721 | 0.00025147 | 0.0015952 | omni3 | Bacteria | Proteobacteria | Alphaproteobacteria | Rhodospirillales | uncultured | uncultured | Azospirillum_sp. |
| OTU_16738 | 0.00028259 | 0.00209368 | pa | Bacteria | Firmicutes | Clostridia | Lachnospirales | Lachnospiraceae | Blautia |  |
| OTU_16738 | 0.00025147 | 0.0015952 | omni3 | Bacteria | Firmicutes | Clostridia | Lachnospirales | Lachnospiraceae | Blautia |  |
| OTU_1692 | 0.00028259 | 0.04274606 | pa | Bacteria | Firmicutes | Clostridia | Lachnospirales | Lachnospiraceae | [Eubacterium]_fissicatena_group | |
| OTU_1692 | 0.00025147 | 0.08946574 | omni3 | Bacteria | Firmicutes | Clostridia | Lachnospirales | Lachnospiraceae | [Eubacterium]_fissicatena_group | |
| OTU_1703 | 0.00028259 | 0.04274606 | pa | Bacteria | Bacteroidota | Bacteroidia | Bacteroidales | Bacteroidaceae | Bacteroides |  |
| OTU_1703 | 0.00025147 | 0.08946574 | omni3 | Bacteria | Bacteroidota | Bacteroidia | Bacteroidales | Bacteroidaceae | Bacteroides |  |
| OTU_1706 | 0.00028259 | 0.00209368 | pa | Bacteria | Firmicutes | Clostridia | Oscillospirales | Oscillospiraceae | Flavonifractor | uncultured_bacterium |
| OTU_1706 | 0.00025147 | 0.0015952 | omni3 | Bacteria | Firmicutes | Clostridia | Oscillospirales | Oscillospiraceae | Flavonifractor | uncultured_bacterium |
| OTU_1759 | 0.00028259 | 0.00209368 | pa | Bacteria | Bacteroidota | Bacteroidia | Bacteroidales | Bacteroidaceae | Bacteroides |  |
| OTU_1759 | 0.00025147 | 0.0015952 | omni3 | Bacteria | Bacteroidota | Bacteroidia | Bacteroidales | Bacteroidaceae | Bacteroides |  |
| OTU_1794 | 0.00028259 | 0.0438027 | pa | Bacteria | Firmicutes | Clostridia | Lachnospirales | Lachnospiraceae | |  |
| OTU_1794 | 0.00025147 | 0.09594416 | omni3 | Bacteria | Firmicutes | Clostridia | Lachnospirales | Lachnospiraceae | |  |
| OTU_1816 | 0.00028259 | 0.0438027 | pa | Bacteria | Verrucomicrobiota | Verrucomicrobiae | Verrucomicrobiales | Akkermansiaceae | Akkermansia | uncultured_bacterium |
| OTU_1816 | 0.00025147 | 0.08946574 | omni3 | Bacteria | Verrucomicrobiota | Verrucomicrobiae | Verrucomicrobiales | Akkermansiaceae | Akkermansia | uncultured_bacterium |
| OTU_1858 | 0.00028259 | 0.0438027 | pa | Bacteria | Bacteroidota | Bacteroidia | Bacteroidales | Bacteroidaceae | Bacteroides |  |
| OTU_1858 | 0.00025147 | 0.08946574 | omni3 | Bacteria | Bacteroidota | Bacteroidia | Bacteroidales | Bacteroidaceae | Bacteroides |  |
| OTU_1867 | 0.00028259 | 0.07595646 | pa | Bacteria | Bacteroidota | Bacteroidia | Bacteroidales | Bacteroidaceae | Bacteroides |  |
| OTU_1867 | 0.00025147 | 0.1881168 | omni3 | Bacteria | Bacteroidota | Bacteroidia | Bacteroidales | Bacteroidaceae | Bacteroides |  |
| OTU_1888 | 0.00028259 | 0.00209368 | pa | Bacteria | Firmicutes | Bacilli | Erysipelotrichales | Erysipelatoclostridiaceae | Erysipelatoclostridium | [Clostridium]_saccharogumia |
| OTU_1888 | 0.00025147 | 0.0015952 | omni3 | Bacteria | Firmicutes | Bacilli | Erysipelotrichales | Erysipelatoclostridiaceae | Erysipelatoclostridium | [Clostridium]_saccharogumia |
| OTU_191 | 0.09003429 | 0.17759926 | omni3 | Bacteria | Firmicutes | Clostridia | Lachnospirales | Lachnospiraceae | Blautia |  |
| OTU_1936 | 0.00028259 | 0.0438027 | pa | Bacteria | Bacteroidota | Bacteroidia | Bacteroidales | Barnesiellaceae | Barnesiella |  |
| OTU_1936 | 0.00025147 | 0.08946574 | omni3 | Bacteria | Bacteroidota | Bacteroidia | Bacteroidales | Barnesiellaceae | Barnesiella |  |
| OTU_1962 | 0.00028259 | 0.00209368 | pa | Bacteria | Firmicutes | Clostridia | Lachnospirales | Lachnospiraceae | |  |
| OTU_1962 | 0.00025147 | 0.0015952 | omni3 | Bacteria | Firmicutes | Clostridia | Lachnospirales | Lachnospiraceae | |  |
| OTU_2080 | 0.00028259 | 0.00209368 | pa | Bacteria | Firmicutes | Clostridia | Lachnospirales | Lachnospiraceae | |  |
| OTU_2080 | 0.00025147 | 0.0015952 | omni3 | Bacteria | Firmicutes | Clostridia | Lachnospirales | Lachnospiraceae | |  |
| OTU_2084 | 0.00028259 | 0.03989632 | pa | Bacteria | Firmicutes | Clostridia | Lachnospirales | Lachnospiraceae | |  |
| OTU_2084 | 0.00025147 | 0.08946574 | omni3 | Bacteria | Firmicutes | Clostridia | Lachnospirales | Lachnospiraceae | |  |
| OTU_2097 | 0.06532271 | 0.09667143 | omni3 | Bacteria | Firmicutes | Clostridia | Lachnospirales | Lachnospiraceae | [Eubacterium]_eligens_group | uncultured_bacterium |
| OTU_2114 | 0.00028259 | 0.00209368 | pa | Bacteria | Firmicutes | Clostridia | Oscillospirales | Ruminococcaceae | Subdoligranulum | uncultured_bacterium |
| OTU_2114 | 0.00025147 | 0.0015952 | omni3 | Bacteria | Firmicutes | Clostridia | Oscillospirales | Ruminococcaceae | Subdoligranulum | uncultured_bacterium |
| OTU_2141 | 0.00028259 | 0.0438027 | pa | Bacteria | Proteobacteria | Alphaproteobacteria | Rhodospirillales | uncultured | uncultured | gut_metagenome |
| OTU_2141 | 0.00025147 | 0.08946574 | omni3 | Bacteria | Proteobacteria | Alphaproteobacteria | Rhodospirillales | uncultured | uncultured | gut_metagenome |
| OTU_2254 | 0.00028259 | 0.00209368 | pa | Bacteria | Actinobacteriota | Actinobacteria | Micrococcales | Micrococcaceae | Rothia |  |
| OTU_2254 | 0.00025147 | 0.0015952 | omni3 | Bacteria | Actinobacteriota | Actinobacteria | Micrococcales | Micrococcaceae | Rothia |  |
| OTU_2346 | 0.00028259 | 0.13340106 | pa | Bacteria | Bacteroidota | Bacteroidia | Bacteroidales | Rikenellaceae | Alistipes |  |
| OTU_2357 | 0.00028259 | 0.00209368 | pa | Bacteria | Verrucomicrobiota | Lentisphaeria | Victivallales | Victivallaceae | Victivallis |  |
| OTU_2357 | 0.00025147 | 0.0015952 | omni3 | Bacteria | Verrucomicrobiota | Lentisphaeria | Victivallales | Victivallaceae | Victivallis |  |
| OTU_2376 | 0.00028259 | 0.04396738 | pa | Bacteria | Bacteroidota | Bacteroidia | Bacteroidales | Bacteroidaceae | Bacteroides |  |
| OTU_2376 | 0.00025147 | 0.09210639 | omni3 | Bacteria | Bacteroidota | Bacteroidia | Bacteroidales | Bacteroidaceae | Bacteroides |  |
| OTU_2415 | 0.00028259 | 0.00209368 | pa | Bacteria | Firmicutes | Clostridia | Lachnospirales | Lachnospiraceae | |  |
| OTU_2415 | 0.00025147 | 0.0015952 | omni3 | Bacteria | Firmicutes | Clostridia | Lachnospirales | Lachnospiraceae | |  |
| OTU_2416 | 0.00028259 | 0.00209368 | pa | Bacteria | Firmicutes | Clostridia | Lachnospirales | Lachnospiraceae | |  |
| OTU_2416 | 0.00025147 | 0.0015952 | omni3 | Bacteria | Firmicutes | Clostridia | Lachnospirales | Lachnospiraceae | |  |
| OTU_2447 | 0.00028259 | 0.00209368 | pa | Bacteria | Firmicutes | Clostridia | Lachnospirales | Lachnospiraceae | Blautia |  |
| OTU_2447 | 0.00025147 | 0.0015952 | omni3 | Bacteria | Firmicutes | Clostridia | Lachnospirales | Lachnospiraceae | Blautia |  |
| OTU_2597 | 0.48762206 | 0.63134615 | freq | Bacteria | Firmicutes | Clostridia | Oscillospirales | Ruminococcaceae | Faecalibacterium | |
| OTU_2597 | 0.01427824 | 0.13095333 | omni3 | Bacteria | Firmicutes | Clostridia | Oscillospirales | Ruminococcaceae | Faecalibacterium | |
| OTU_260 | 0.00028259 | 0.0438027 | pa | Bacteria | Actinobacteriota | Coriobacteriia | Coriobacteriales | Eggerthellaceae | uncultured | uncultured_bacterium |
| OTU_260 | 0.00025147 | 0.08946574 | omni3 | Bacteria | Actinobacteriota | Coriobacteriia | Coriobacteriales | Eggerthellaceae | uncultured | uncultured_bacterium |
| OTU_2711 | 0.00028259 | 0.00209368 | pa | Bacteria | Firmicutes | Clostridia | Lachnospirales | Lachnospiraceae | Blautia |  |
| OTU_2711 | 0.00025147 | 0.0015952 | omni3 | Bacteria | Firmicutes | Clostridia | Lachnospirales | Lachnospiraceae | Blautia |  |
| OTU_2791 | 0.00028259 | 0.04274606 | pa | Bacteria | Firmicutes | Clostridia | Oscillospirales | Ruminococcaceae | Faecalibacterium | |
| OTU_2791 | 0.00025147 | 0.08946574 | omni3 | Bacteria | Firmicutes | Clostridia | Oscillospirales | Ruminococcaceae | Faecalibacterium | |
| OTU_2823 | 0.00028259 | 0.0438027 | pa | Bacteria | Firmicutes | Clostridia | Lachnospirales | Lachnospiraceae | UC5-1-2E3 | uncultured_bacterium |
| OTU_2823 | 0.00025147 | 0.08946574 | omni3 | Bacteria | Firmicutes | Clostridia | Lachnospirales | Lachnospiraceae | UC5-1-2E3 | uncultured_bacterium |
| OTU_2900 | 0.00028259 | 0.03989632 | pa | Bacteria | Firmicutes | Clostridia | Lachnospirales | Lachnospiraceae | |  |
| OTU_2900 | 0.00025147 | 0.08946574 | omni3 | Bacteria | Firmicutes | Clostridia | Lachnospirales | Lachnospiraceae | |  |
| OTU_2903 | 0.00028259 | 0.00209368 | pa | Bacteria | Firmicutes | Clostridia | Lachnospirales | Lachnospiraceae | |  |
| OTU_2903 | 0.00025147 | 0.0015952 | omni3 | Bacteria | Firmicutes | Clostridia | Lachnospirales | Lachnospiraceae | |  |
| OTU_2917 | 0.00028259 | 0.0438027 | pa | Bacteria | Cyanobacteria | Vampirivibrionia | Gastranaerophilales | Gastranaerophilales | Gastranaerophilales | |
| OTU_2917 | 0.00025147 | 0.08946574 | omni3 | Bacteria | Cyanobacteria | Vampirivibrionia | Gastranaerophilales | Gastranaerophilales | Gastranaerophilales | |
| OTU_2927 | 0.00028259 | 0.0438027 | pa | Bacteria | Firmicutes | Clostridia | Lachnospirales | Lachnospiraceae | Anaerostipes | |
| OTU_2927 | 0.00025147 | 0.08946574 | omni3 | Bacteria | Firmicutes | Clostridia | Lachnospirales | Lachnospiraceae | Anaerostipes | |
| OTU_3048 | 0.00028259 | 0.0438027 | pa | Bacteria | Firmicutes | Clostridia | Lachnospirales | Lachnospiraceae | [Eubacterium]_hallii_group | uncultured_bacterium |
| OTU_3048 | 0.00025147 | 0.08984674 | omni3 | Bacteria | Firmicutes | Clostridia | Lachnospirales | Lachnospiraceae | [Eubacterium]_hallii_group | uncultured_bacterium |
| OTU_3122 | 0.00028259 | 0.00209368 | pa | Bacteria | Firmicutes | Clostridia | Oscillospirales | Ruminococcaceae | Incertae_Sedis | [Clostridium]_leptum |
| OTU_3122 | 0.00025147 | 0.0015952 | omni3 | Bacteria | Firmicutes | Clostridia | Oscillospirales | Ruminococcaceae | Incertae_Sedis | [Clostridium]_leptum |
| OTU_3327 | 0.00028259 | 0.00209368 | pa | Bacteria | Bacteroidota | Bacteroidia | Bacteroidales | Rikenellaceae | Alistipes | Alistipes_finegoldii |
| OTU_3327 | 0.00025147 | 0.0015952 | omni3 | Bacteria | Bacteroidota | Bacteroidia | Bacteroidales | Rikenellaceae | Alistipes | Alistipes_finegoldii |
| OTU_3406 | 0.00028259 | 0.00209368 | pa | Bacteria | Firmicutes | Clostridia | Lachnospirales | Lachnospiraceae | |  |
| OTU_3406 | 0.00025147 | 0.0015952 | omni3 | Bacteria | Firmicutes | Clostridia | Lachnospirales | Lachnospiraceae | |  |
| OTU_3463 | 0.00028259 | 0.0438027 | pa | Bacteria | Firmicutes | Clostridia | Lachnospirales | Lachnospiraceae | [Eubacterium]_hallii_group | |
| OTU_3463 | 0.00025147 | 0.08946574 | omni3 | Bacteria | Firmicutes | Clostridia | Lachnospirales | Lachnospiraceae | [Eubacterium]_hallii_group | |
| OTU_3511 | 0.00028259 | 0.00209368 | pa | Bacteria | Firmicutes | Clostridia | Lachnospirales | Lachnospiraceae | Lachnoclostridium | |
| OTU_3511 | 0.00025147 | 0.0015952 | omni3 | Bacteria | Firmicutes | Clostridia | Lachnospirales | Lachnospiraceae | Lachnoclostridium | |
| OTU_3542 | 0.00028259 | 0.0438027 | pa | Bacteria | Firmicutes | Clostridia | Oscillospirales | Ruminococcaceae | Ruminococcus | Ruminococcus_bicirculans |
| OTU_3542 | 0.00025147 | 0.08946574 | omni3 | Bacteria | Firmicutes | Clostridia | Oscillospirales | Ruminococcaceae | Ruminococcus | Ruminococcus_bicirculans |
| OTU_3630 | 0.00028259 | 0.04859552 | pa | Bacteria | Firmicutes | Clostridia | Lachnospirales | Lachnospiraceae | |  |
| OTU_3630 | 0.00025147 | 0.1062766 | omni3 | Bacteria | Firmicutes | Clostridia | Lachnospirales | Lachnospiraceae | |  |
| OTU_3814 | 0.06252473 | 0.13186224 | omni3 | Bacteria | Bacteroidota | Bacteroidia | Bacteroidales | Tannerellaceae | Parabacteroides | |
| OTU_3851 | 0.00028259 | 0.00209368 | pa | Bacteria | Firmicutes | Clostridia | Lachnospirales | Lachnospiraceae | [Ruminococcus]_torques_group | |
| OTU_3851 | 0.00025147 | 0.0015952 | omni3 | Bacteria | Firmicutes | Clostridia | Lachnospirales | Lachnospiraceae | [Ruminococcus]_torques_group | |
| OTU_389 | 0.48762206 | 0.23350915 | freq | Bacteria | Firmicutes | Clostridia | Lachnospirales | Lachnospiraceae | [Ruminococcus]_torques_group | |
| OTU_389 | 0.01646495 | 0.03282922 | omni3 | Bacteria | Firmicutes | Clostridia | Lachnospirales | Lachnospiraceae | [Ruminococcus]_torques_group | |
| OTU_3943 | 0.00028259 | 0.04302184 | pa | Bacteria | Proteobacteria | Alphaproteobacteria | Rhodospirillales | uncultured | uncultured | Azospirillum_sp. |
| OTU_3943 | 0.00025147 | 0.08946574 | omni3 | Bacteria | Proteobacteria | Alphaproteobacteria | Rhodospirillales | uncultured | uncultured | Azospirillum_sp. |
| OTU_3973 | 0.00028259 | 0.0438027 | pa | Bacteria | Firmicutes | Clostridia | Oscillospirales | Ruminococcaceae | Ruminococcus | |
| OTU_3973 | 0.00025147 | 0.08946574 | omni3 | Bacteria | Firmicutes | Clostridia | Oscillospirales | Ruminococcaceae | Ruminococcus | |
| OTU_4162 | 0.00028259 | 0.00209368 | pa | Bacteria | Firmicutes | Clostridia | Oscillospirales | Ruminococcaceae | Candidatus_Soleaferrea | Ruminococcaceae_bacterium |
| OTU_4162 | 0.00025147 | 0.0015952 | omni3 | Bacteria | Firmicutes | Clostridia | Oscillospirales | Ruminococcaceae | Candidatus_Soleaferrea | Ruminococcaceae_bacterium |
| OTU_42 | 0.00028259 | 0.15503056 | pa | Bacteria | Proteobacteria | Gammaproteobacteria | Burkholderiales | Sutterellaceae | Sutterella |  |
| OTU_4219 | 0.00028259 | 0.00209368 | pa | Bacteria | Firmicutes | Clostridia | Lachnospirales | Lachnospiraceae | |  |
| OTU_4219 | 0.00025147 | 0.0015952 | omni3 | Bacteria | Firmicutes | Clostridia | Lachnospirales | Lachnospiraceae | |  |
| OTU_425 | 0.00028259 | 0.0438027 | pa | Bacteria | Firmicutes | Clostridia | Lachnospirales | Lachnospiraceae | |  |
| OTU_425 | 0.00025147 | 0.08984674 | omni3 | Bacteria | Firmicutes | Clostridia | Lachnospirales | Lachnospiraceae | |  |
| OTU_4287 | 0.01953753 | 0.23038596 | pa | Bacteria | Bacteroidota | Bacteroidia | Bacteroidales | Rikenellaceae | Alistipes |  |
| OTU_43 | 0.02060222 | 0.2572884 | pa | Bacteria | Firmicutes | Clostridia | Lachnospirales | Lachnospiraceae | Lachnospiraceae_ND3007_group | uncultured_bacterium |
| OTU_4315 | 0.00028259 | 0.00209368 | pa | Bacteria | Firmicutes | Clostridia | Lachnospirales | Lachnospiraceae | |  |
| OTU_4315 | 0.00025147 | 0.0015952 | omni3 | Bacteria | Firmicutes | Clostridia | Lachnospirales | Lachnospiraceae | |  |
| OTU_4332 | 0.00028259 | 0.00209368 | pa | Bacteria | Firmicutes | Clostridia | Oscillospirales | Ruminococcaceae | |  |
| OTU_4332 | 0.00025147 | 0.0015952 | omni3 | Bacteria | Firmicutes | Clostridia | Oscillospirales | Ruminococcaceae | |  |
| OTU_4346 | 0.00028259 | 0.00209368 | pa | Bacteria | Bacteroidota | Bacteroidia | Bacteroidales | Tannerellaceae | Parabacteroides | |
| OTU_4346 | 0.00025147 | 0.0015952 | omni3 | Bacteria | Bacteroidota | Bacteroidia | Bacteroidales | Tannerellaceae | Parabacteroides | |
| OTU_4456 | 0.00028259 | 0.03077716 | pa | Bacteria | Bacteroidota | Bacteroidia | Bacteroidales | Bacteroidaceae | Bacteroides |  |
| OTU_4456 | 0.00025147 | 0.08669188 | omni3 | Bacteria | Bacteroidota | Bacteroidia | Bacteroidales | Bacteroidaceae | Bacteroides |  |
| OTU_45 | 0.00028259 | 0.209375 | pa | Bacteria | Proteobacteria | Gammaproteobacteria | Enterobacterales | Enterobacteriaceae | Escherichia-Shigella | |
| OTU_4505 | 0.00028259 | 0.04274606 | pa | Bacteria | Firmicutes | Clostridia | Lachnospirales | Lachnospiraceae | |  |
| OTU_4505 | 0.00025147 | 0.08946574 | omni3 | Bacteria | Firmicutes | Clostridia | Lachnospirales | Lachnospiraceae | |  |
| OTU_452 | 0.48762206 | 0.60796296 | freq | Bacteria | Firmicutes | Clostridia | Lachnospirales | Lachnospiraceae | Sellimonas | uncultured_bacterium |
| OTU_452 | 0.01427824 | 0.08946574 | omni3 | Bacteria | Firmicutes | Clostridia | Lachnospirales | Lachnospiraceae | Sellimonas | uncultured_bacterium |
| OTU_4613 | 0.00028259 | 0.00209368 | pa | Bacteria | Bacteroidota | Bacteroidia | Bacteroidales | Bacteroidaceae | Bacteroides |  |
| OTU_4613 | 0.00025147 | 0.0015952 | omni3 | Bacteria | Bacteroidota | Bacteroidia | Bacteroidales | Bacteroidaceae | Bacteroides |  |
| OTU_4637 | 0.00028259 | 0.00209368 | pa | Bacteria | Verrucomicrobiota | Verrucomicrobiae | Verrucomicrobiales | Akkermansiaceae | Akkermansia | uncultured_bacterium |
| OTU_4637 | 0.00025147 | 0.0015952 | omni3 | Bacteria | Verrucomicrobiota | Verrucomicrobiae | Verrucomicrobiales | Akkermansiaceae | Akkermansia | uncultured_bacterium |
| OTU_4724 | 0.00028259 | 0.03620843 | pa | Bacteria | Firmicutes | Clostridia | Lachnospirales | Lachnospiraceae | |  |
| OTU_4724 | 0.00025147 | 0.08669188 | omni3 | Bacteria | Firmicutes | Clostridia | Lachnospirales | Lachnospiraceae | |  |
| OTU_4762 | 0.00028259 | 0.00209368 | pa | Bacteria | Firmicutes | Clostridia | Lachnospirales | Lachnospiraceae | [Ruminococcus]_torques_group | |
| OTU_4762 | 0.00025147 | 0.0015952 | omni3 | Bacteria | Firmicutes | Clostridia | Lachnospirales | Lachnospiraceae | [Ruminococcus]_torques_group | |
| OTU_485 | 0.00028259 | 0.0438027 | pa | Bacteria | Firmicutes | Clostridia | Peptostreptococcales-Tissierellales | Anaerovoracaceae | [Eubacterium]_nodatum_group | uncultured_bacterium |
| OTU_485 | 0.00025147 | 0.11644138 | omni3 | Bacteria | Firmicutes | Clostridia | Peptostreptococcales-Tissierellales | Anaerovoracaceae | [Eubacterium]_nodatum_group | uncultured_bacterium |
| OTU_495 | 0.06743218 | 0.10382669 | omni3 | Bacteria | Bacteroidota | Bacteroidia | Bacteroidales | Bacteroidaceae | Bacteroides | Bacteroides_coprophilus |
| OTU_532 | 0.00028259 | 0.00209368 | pa | Bacteria | Firmicutes | Clostridia | Oscillospirales | Oscillospiraceae | Colidextribacter | |
| OTU_532 | 0.00025147 | 0.0015952 | omni3 | Bacteria | Firmicutes | Clostridia | Oscillospirales | Oscillospiraceae | Colidextribacter | |
| OTU_5682 | 0.00028259 | 0.0438027 | pa | Bacteria | Bacteroidota | Bacteroidia | Bacteroidales | Bacteroidaceae | Bacteroides |  |
| OTU_5682 | 0.00025147 | 0.09382654 | omni3 | Bacteria | Bacteroidota | Bacteroidia | Bacteroidales | Bacteroidaceae | Bacteroides |  |
| OTU_572 | 0.00028259 | 0.0438027 | pa | Bacteria | Firmicutes | Bacilli | Lactobacillales | Lactobacillaceae | Lactobacillus | |
| OTU_572 | 0.00025147 | 0.08946574 | omni3 | Bacteria | Firmicutes | Bacilli | Lactobacillales | Lactobacillaceae | Lactobacillus | |
| OTU_5814 | 0.00028259 | 0.0438027 | pa | Bacteria | Firmicutes | Clostridia | Lachnospirales | Lachnospiraceae | Roseburia |  |
| OTU_5814 | 0.00025147 | 0.09667143 | omni3 | Bacteria | Firmicutes | Clostridia | Lachnospirales | Lachnospiraceae | Roseburia |  |
| OTU_637 | 0.00028259 | 0.0438027 | pa | Bacteria | Bacteroidota | Bacteroidia | Bacteroidales | Bacteroidaceae | Bacteroides |  |
| OTU_637 | 0.00025147 | 0.08946574 | omni3 | Bacteria | Bacteroidota | Bacteroidia | Bacteroidales | Bacteroidaceae | Bacteroides |  |
| OTU_64 | 0.00028259 | 0.20493879 | pa | Bacteria | Bacteroidota | Bacteroidia | Bacteroidales | Barnesiellaceae | Barnesiella |  |
| OTU_647 | 0.00028259 | 0.00209368 | pa | Bacteria | Bacteroidota | Bacteroidia | Bacteroidales | Barnesiellaceae | Barnesiella |  |
| OTU_647 | 0.00025147 | 0.0015952 | omni3 | Bacteria | Bacteroidota | Bacteroidia | Bacteroidales | Barnesiellaceae | Barnesiella |  |
| OTU_651 | 0.00028259 | 0.04396738 | pa | Bacteria | Verrucomicrobiota | Lentisphaeria | Victivallales | Victivallaceae | Victivallis | Victivallis_vadensis |
| OTU_651 | 0.00025147 | 0.09594416 | omni3 | Bacteria | Verrucomicrobiota | Lentisphaeria | Victivallales | Victivallaceae | Victivallis | Victivallis_vadensis |
| OTU_68 | 0.48762206 | 0.63134615 | freq | Bacteria | Bacteroidota | Bacteroidia | Bacteroidales | Rikenellaceae | Alistipes | Alistipes_shahii |
| OTU_68 | 0.0121694 | 0.08984674 | omni3 | Bacteria | Bacteroidota | Bacteroidia | Bacteroidales | Rikenellaceae | Alistipes | Alistipes_shahii |
| OTU_684 | 0.02074305 | 0.04444665 | omni3 | Bacteria | Firmicutes | Clostridia | Lachnospirales | Lachnospiraceae | Agathobacter | |
| OTU_7180 | 0.00028259 | 0.06907086 | pa | Bacteria | Firmicutes | Bacilli | Erysipelotrichales | Erysipelatoclostridiaceae | Erysipelotrichaceae_UCG-003 | |
| OTU_7180 | 0.00025147 | 0.16177101 | omni3 | Bacteria | Firmicutes | Bacilli | Erysipelotrichales | Erysipelatoclostridiaceae | Erysipelotrichaceae_UCG-003 | |
| OTU_7692 | 0.00028259 | 0.00209368 | pa | Bacteria | Firmicutes | Clostridia | Lachnospirales | Lachnospiraceae | |  |
| OTU_7692 | 0.00025147 | 0.0015952 | omni3 | Bacteria | Firmicutes | Clostridia | Lachnospirales | Lachnospiraceae | |  |
| OTU_798 | 0.00028259 | 0.05338896 | pa | Bacteria | Bacteroidota | Bacteroidia | Bacteroidales | Barnesiellaceae | Coprobacter | Coprobacter_secundus |
| OTU_798 | 0.00025147 | 0.12272897 | omni3 | Bacteria | Bacteroidota | Bacteroidia | Bacteroidales | Barnesiellaceae | Coprobacter | Coprobacter_secundus |
| OTU_851 | 0.00028259 | 0.0438027 | pa | Bacteria | Bacteroidota | Bacteroidia | Bacteroidales | Bacteroidaceae | Bacteroides |  |
| OTU_851 | 0.00025147 | 0.08946574 | omni3 | Bacteria | Bacteroidota | Bacteroidia | Bacteroidales | Bacteroidaceae | Bacteroides |  |
| OTU_8520 | 0.00028259 | 0.00209368 | pa | Bacteria | Bacteroidota | Bacteroidia | Bacteroidales | Bacteroidaceae | Bacteroides |  |
| OTU_8520 | 0.00025147 | 0.0015952 | omni3 | Bacteria | Bacteroidota | Bacteroidia | Bacteroidales | Bacteroidaceae | Bacteroides |  |
| OTU_8769 | 0.00028259 | 0.06565795 | pa | Bacteria | Firmicutes | Clostridia | Lachnospirales | Lachnospiraceae | |  |
| OTU_8769 | 0.00025147 | 0.17661187 | omni3 | Bacteria | Firmicutes | Clostridia | Lachnospirales | Lachnospiraceae | |  |
| OTU_885 | 0.00028259 | 0.0438027 | pa | Bacteria | Bacteroidota | Bacteroidia | Bacteroidales | Bacteroidaceae | Bacteroides |  |
| OTU_885 | 0.00025147 | 0.08984674 | omni3 | Bacteria | Bacteroidota | Bacteroidia | Bacteroidales | Bacteroidaceae | Bacteroides |  |
| OTU_903 | 0.00028259 | 0.03748501 | pa | Bacteria | Firmicutes | Clostridia | Lachnospirales | Lachnospiraceae | Blautia | Blautia_hydrogenotrophica |
| OTU_903 | 0.00025147 | 0.08669188 | omni3 | Bacteria | Firmicutes | Clostridia | Lachnospirales | Lachnospiraceae | Blautia | Blautia_hydrogenotrophica |
| OTU_908 | 0.00028259 | 0.04859552 | pa | Bacteria | Firmicutes | Clostridia | Lachnospirales | Lachnospiraceae | Roseburia |  |
| OTU_908 | 0.00025147 | 0.09382654 | omni3 | Bacteria | Firmicutes | Clostridia | Lachnospirales | Lachnospiraceae | Roseburia |  |
| OTU_932 | 0.00028259 | 0.07645899 | pa | Bacteria | Bacteroidota | Bacteroidia | Bacteroidales | Bacteroidaceae | Bacteroides |  |
| OTU_932 | 0.00025147 | 0.17782917 | omni3 | Bacteria | Bacteroidota | Bacteroidia | Bacteroidales | Bacteroidaceae | Bacteroides |  |
| OTU_9343 | 0.00028259 | 0.00209368 | pa | Bacteria | Firmicutes | Clostridia | Clostridia_UCG-014 | Clostridia_UCG-014 | Clostridia_UCG-014 | |
| OTU_9343 | 0.00025147 | 0.0015952 | omni3 | Bacteria | Firmicutes | Clostridia | Clostridia_UCG-014 | Clostridia_UCG-014 | Clostridia_UCG-014 | |
| OTU_954 | 0.00028259 | 0.07141106 | pa | Bacteria | Bacteroidota | Bacteroidia | Bacteroidales | Bacteroidaceae | Bacteroides |  |
| OTU_954 | 0.00025147 | 0.17759926 | omni3 | Bacteria | Bacteroidota | Bacteroidia | Bacteroidales | Bacteroidaceae | Bacteroides |  |
| OTU_9719 | 0.00028259 | 0.00209368 | pa | Bacteria | Firmicutes | Clostridia | Lachnospirales | Lachnospiraceae | Roseburia |  |
| OTU_9719 | 0.00025147 | 0.0015952 | omni3 | Bacteria | Firmicutes | Clostridia | Lachnospirales | Lachnospiraceae | Roseburia |  |
| OTU_975 | 0.00028259 | 0.04915797 | pa | Bacteria | Firmicutes | Clostridia | Lachnospirales | Lachnospiraceae | |  |
| OTU_975 | 0.00025147 | 0.10042118 | omni3 | Bacteria | Firmicutes | Clostridia | Lachnospirales | Lachnospiraceae | |  |
| OTU_981 | 0.00028259 | 0.05182409 | pa | Bacteria | Firmicutes | Clostridia | Lachnospirales | Lachnospiraceae | Lachnoclostridium | [Clostridium]_scindens |
| OTU_981 | 0.00025147 | 0.11725 | omni3 | Bacteria | Firmicutes | Clostridia | Lachnospirales | Lachnospiraceae | Lachnoclostridium | [Clostridium]_scindens |

**Supplementary Figure S5**: Mediating OTUs showing relative abundance for MAP+ and MAP- cohorts.

**References**

1. Dixon P. VEGAN, a package of R functions for community ecology. Journal of vegetation science. 2003;14(6):927-30.

2. McMurdie PJ, Holmes S. phyloseq: an R package for reproducible interactive analysis and graphics of microbiome census data. PloS one. 2013;8(4):e61217.

3. Zhang Y, Jing G, Chen Y, Li J, Su X. Hierarchical Meta-Storms enables comprehensive and rapid comparison of microbiome functional profiles on a large scale using hierarchical dissimilarity metrics and parallel computing. Bioinformatics Advances. 2021;1(1):vbab003.

4. Tang Z-Z, Chen G. Robust and powerful differential composition tests for clustered microbiome data. Statistics in Biosciences. 2021;13:200-16.

5. Susin A, Wang Y, Lê Cao K-A, Calle ML. Variable selection in microbiome compositional data analysis. NAR Genomics and Bioinformatics. 2020;2(2):lqaa029.

6. Salama DM. Microbial Community Profiling of Leafy Green Vegetables Using Multi-Omics Strategy: University of Nebraska at Omaha; 2023.

7. Clarke K, Ainsworth M. A method of linking multivariate community structure to environmental variables. Marine Ecology-Progress Series. 1993;92:205-.

8. Taylor M. sinkr: A Collection of Functions Featured on the Blog'me nugget'. R package version 10. 2014.

9. Shade A, Stopnisek N. Abundance-occupancy distributions to prioritize plant core microbiome membership. Current opinion in microbiology. 2019;49:50-8.

10. Foster ZS, Sharpton TJ, Grünwald NJ. Metacoder: An R package for visualization and manipulation of community taxonomic diversity data. PLoS computational biology. 2017;13(2):e1005404.

11. Yue Y, Hu Y-J. A new approach to testing mediation of the microbiome at both the community and individual taxon levels. Bioinformatics. 2022;38(12):3173-80.

12. Wei T, Simko V, Levy M, Xie Y, Jin Y, Zemla J. Package ‘corrplot’. Statistician. 2017;56(316):e24.
